# Testing the Efficacy of a Brief, Self-Guided Mindfulness Ecological Momentary Intervention on Emotion Regulation and Self-Compassion in Social Anxiety Disorder: Randomized Controlled Trial

**DOI:** 10.2196/53712

**Published:** 2024-04-19

**Authors:** Nur Hani Zainal, Hui Han Tan, Ryan Yee Shiun Hong, Michelle Gayle Newman

**Affiliations:** 1 Department of Psychology National University of Singapore Singapore Singapore; 2 Department of Health Policy Harvard Medical School Boston, MA United States; 3 Department of Psychology The Pennsylvania State University University Park, PA United States

**Keywords:** social anxiety disorder, mindfulness, ecological momentary intervention, randomized controlled trial, emotion regulation, self-compassion, mechanisms of change, mobile phone, momentary interventions, self-monitoring app, regulations, participant

## Abstract

**Background:**

Theories propose that brief, mobile, self-guided mindfulness ecological momentary interventions (MEMIs) could enhance emotion regulation (ER) and self-compassion. Such changes are posited to be mechanisms of change. However, rigorous tests of these theories have not been conducted.

**Objective:**

In this assessor-blinded, parallel-group randomized controlled trial, we aimed to test these theories in social anxiety disorder (SAD).

**Methods:**

Participants with SAD (defined as having a prerandomization cut-off score ≥20 on the Social Phobia Inventory self-report) were randomized to a 14-day fully self-guided MEMI (96/191, 50.3%) or self-monitoring app (95/191, 49.7%) arm. They completed web-based self-reports of 6 clinical outcome measures at prerandomization, 15-day postintervention (administered the day after the intervention ended), and 1-month follow-up time points. ER and self-compassion were assessed at preintervention and 7-day midintervention time points. Multilevel modeling determined the efficacy of MEMI on ER and self-compassion domains from pretrial to midintervention time points. Bootstrapped parallel multilevel mediation analysis examined the mediating role of pretrial to midintervention ER and self-compassion domains on the efficacy of MEMI on 6 clinical outcomes.

**Results:**

Participants demonstrated strong compliance, with 78% (149/191) engaging in at least 80% of the MEMI and self-monitoring prompts. MEMI was more efficacious than the self-monitoring app in decreasing ER goal–directed behavior difficulties (between-group Cohen *d*=−0.24) and lack of emotional clarity (Cohen *d*=0.16) and increasing self-compassion social connectedness (Cohen *d*=0.19), nonidentification with emotions (Cohen *d*=0.16), and self-kindness (Cohen *d*=0.19) from pretrial to midintervention time points. The within-group effect sizes from pretrial to midintervention were larger in the MEMI arm than in the self-monitoring app arm (ER goal–directed behavior difficulties: Cohen *d*=−0.73 vs −0.29, lack of emotional clarity: Cohen *d*=−0.39 vs −0.21, self-compassion domains of social connectedness: Cohen *d*=0.45 vs 0.19, nonidentification with emotions: Cohen *d*=0.63 vs 0.48, and self-kindness: Cohen *d*=0.36 vs 0.10). Self-monitoring, but not MEMI, alleviated ER emotional awareness issues (between-group Cohen *d*=0.11 and within-group: Cohen *d*=−0.29 vs −0.13) and reduced self-compassion acknowledging shared human struggles (between-group Cohen *d*=0.26 and within-group: Cohen *d*=−0.23 vs 0.13). No ER and self-compassion domains were mediators of the effect of MEMI on SAD symptoms (*P*=.07-<.99), generalized anxiety symptoms (*P*=.16-.98), depression severity (*P*=.20-.94), repetitive negative thinking (*P*=.12-.96), and trait mindfulness (*P*=.18-.99) from pretrial to postintervention time points. Similar nonsignificant mediation effects emerged for all of these clinical outcomes from pretrial to 1-month follow-up time points (*P*=.11-.98).

**Conclusions:**

Brief, fully self-guided, mobile MEMIs efficaciously increased specific self-compassion domains and decreased ER difficulties associated with goal pursuit and clarity of emotions from pretrial to midintervention time points. Higher-intensity MEMIs may be required to pinpoint the specific change mechanisms in ER and self-compassion domains of SAD.

**Trial Registration:**

Open Science Framework (OSF) Registries; osf.io/m3kxz https://osf.io/m3kxz

## Introduction

### Background

Emotion regulation (ER) has been defined as a deliberate action taken to modulate emotional states [1]. Situations can trigger emotional reactions, including short- and long-term emotional responses [2]. Persistent suboptimal behaviors that limit opportunities and quality of life are inherent to various emotional disorders, such as avoidance of feared situations in social anxiety disorder (SAD) [3], brooding in depression [4], and impulsivity in attention-deficit/hyperactivity disorder [5]. Elevating self-compassion could facilitate better ER by fostering discomfort tolerance [6]. Self-compassion is described as accepting one’s experience without judgment and showing oneself kindness [7-9]. Increased self-judgment might create anxiety and depression via emotional dysregulation. Such anxiety and depression could lead to avoidance as a way to manage negative affect [10]. Chronic avoidance of social situations and failure to optimally face challenges head-on can hinder one from building self-efficacy; decrease pleasure derived from recreational activities; and restrict work, school, and other life opportunities [11-13]. Therefore, developing efficacious interventions to improve ER and self-compassion is essential.

Mindfulness-based interventions (MBIs) might enhance ER and self-compassion simultaneously. The monitoring and acceptance theory proposed that MBIs could boost ER by increasing acceptance, curiosity, and equanimity, coinciding with better interpersonal functioning [14]. This theory suggests that enhancing various outcomes in MBIs relies on integrating attention monitoring and acceptance skills [15]. It proposes that acceptance functions as an ER skill that promotes nonreactivity, self-compassion, and receptiveness to current experiences. The omission of acceptance skills training could potentially negate the stress-buffering and social cognitive advantages of MBIs. Consistent with the monitoring and acceptance theory, meta-analyses have shown inverse relations among self-compassion and anxiety, depressive symptoms, and stress [16] and found that MBIs were superior to controls in improving mental health symptoms and self-compassion [17]. However, traditional in-person MBIs, such as mindfulness-based stress reduction [18] and mindfulness-based cognitive therapy [19], typically involve significant financial costs, time commitments, and potential travel requirements, often extending from 1 hour to 2.5 hours per week across 8 to 16 sessions with 6-hour retreat sessions [20]. Individuals with high levels of emotional dysregulation and self-compassion deficits tend to self-report shame, stigma, and other hindrances to treatment seeking [21,22]. Those experiencing these struggles may not seek in-person therapy or may not remain in treatment, fearing the negative emotions associated with the treatment process. Collectively, such research emphasizes the importance of developing scalable (eg, app based) and concise MBIs.

Concise and scalable MBIs, typically lasting up to 2 weeks [23], may enhance ER and self-compassion. For instance, enhanced ER skills and reduced stress levels were observed among smokers and nonsmokers after a 5-day MBI, compared to ER and stress observed before the intervention [24]. Similarly, a single-session MBI was associated with increased cognitive reappraisal and reduced emotional suppression (ER domains) among graduate students over 3 months [25]. However, the absence of a control arm in these studies precluded knowing whether the brief MBI would outperform an active control, an essential aspect for drawing causal inferences [26]. Another study, a 14-day MBI randomized controlled trial (RCT) [27], implied this possibility by showing that acceptance was critical in effectively reducing loneliness and enhancing social engagement in everyday experiences. Another RCT showed that a single-session MBI was linked to better ER in trauma-distressed people [28]. Relatedly, 5-session MBIs consistently outperformed controls, raising self-compassion and mindfulness among undergraduates [29,30]. Together, these studies suggested that brief mindfulness ecological momentary interventions (MEMIs) that repeatedly instructed mindfulness exercises and recorded symptom levels in real time could outperform an active control in enhancing ER and self-compassion over time in clinical samples.

Moreover, improved ER and self-regulation may be a theoretical change mechanism explaining why brief, fully self-guided mobile MEMIs conferred mental health benefits such as symptom reduction over time [31,32]. Such theories posit that brief, fully self-guided mobile MEMIs may enhance the capacity to observe internal reactions in emotionally charged situations, enabling individuals to recognize when they are caught in their emotions, to pause to regain composure before reacting, and to practice self-kindness. Indeed, 2 recent experiments of diverse mindfulness practices showed that state-level and trait-level self-compassion improvements were linked to increased self-guided mindfulness practices and quality of life across 14 days in nonclinical samples [33]. Relatedly, baseline higher trait observing and describing mindfulness facets predicted reduced anxiety and depression via enhanced ER across an internet-delivered 8-week MBI in healthy adults [34]. This finding might extend to brief, fully self-guided mobile MEMIs for clinical samples. The dearth of mediation analyses testing the mechanistic role of ER and self-compassion highlights the importance of conducting such studies, particularly among individuals with emotional disorders. Discovering treatment mediators (proxy change mechanisms) might aid with fine-tuning and refining existing brief, scalable MEMIs for clinical samples [35].

SAD presents as one potential clinical sample case in point. Fostering better ER and self-compassion as treatment targets via brief, fully self-guided, mobile MEMIs could be especially advantageous for individuals with SAD, as they tend to exhibit poor ER, such as excessive avoidance and difficulties applying cognitive modification techniques [36]. Teaching nonjudgment and nonreactivity skills in SAD via the MEMI might pave the way for fostering cognitive modification and other ER skills and promote active efforts to increase exposure to various interpersonal situations. Furthermore, self-compassion has shown inverse associations with overall SAD symptoms and specific cognitive processes, such as the fear of both negative and positive evaluation [37] and postevent ruminative processing [38]. Those with versus without SAD felt it was essential to control their emotions, did not believe in emotion malleability [39], and had trouble practicing acceptance of emotional responses [40]. Therefore, MEMIs instructing present-moment focus and acceptance of myriad emotional experiences could remedy these psychological rigidities by enhancing ER skills, thereby improving a wide array of responses to fear-inducing social situations. Construed clinically, these studies suggest that individuals with SAD have room for growth in terms of benefiting from brief, fully self-guided, mobile MEMIs to reduce various ER difficulties and enhance diverse self-compassion domains across time.

Such ER difficulties could pertain to *nonacceptance of emotions* (proclivity to exhibit nonaccepting responses to one’s distress) and *emotional awareness issues* (the inclination to focus upon and recognize emotions within oneself). They might also be linked to a *lack of emotional clarity* (lucidity regarding present-moment emotions) and *goal-directed behavior difficulties* (maintaining focus and achieving tasks during episodes of negative emotional states). Furthermore, ER difficulties could encompass *impulse control issues* (inability to sustain behavioral self-regulation amid negative emotional states) and *strategy use problems* (lack of conviction for having the ability to effectively regulate emotions when feeling upset [41]). The facets of self-compassion might also pertain to *acknowledging shared human struggles* (acknowledgment of collective human experience, recognizing that all individuals encounter failures, make errors, and navigate imperfect life trajectories) and *social connectedness* (a sense of being linked socially with other humans). Cultivating self-compassion also embraces *mindfulness* (a state of cognizant immersion in one’s immediate experience, characterized by clarity and equilibrium) and *nonidentification with emotions* (absence or lack of tendency to become ensnared in an exaggerated narrative about the adverse facets of one’s self or life experiences). In addition, self-compassion includes *nonjudgment toward oneself* (absence or lack of tendency to be overly self-critical) and *self-kindness* (practice of approaching oneself with support and understanding) [42].

### Objectives

On the basis of the theory and logic outlined, the aims of this study were 2-fold. First, we hypothesized that a 14-day MEMI would be superior to a self-monitoring control condition in reducing various domains of ER difficulties and enhancing self-compassion from pretrial to midintervention time points across 7 days (hypothesis 1). Specifically, we examined the 6 difficulties in ER and self-compassion domains mentioned above as pretrial to midintervention outcomes. Second, we hypothesized that the effect of the 14-day MEMI versus self-monitoring app on change in SAD-related outcomes from pretrial to postintervention time points (pre-post; hypothesis 2a) or prerandomization to 1-month follow-up (1MFU) time points (pre-1MFU; hypothesis 2b) would be mediated via a change in ER difficulties and self-compassion domains assessed from pretrial to midintervention time points. Specifically, the outcomes examined were SAD symptoms, generalized anxiety symptoms, depression severity, repetitive negative thinking, and trait mindfulness. Our study was an extension of a primary RCT, which showed that brief MEMI and self-monitoring app led to sustained changes in all of these clinical outcomes, with small-to-large effect sizes from pre-post and pre-1MFU time points. There were no differences between MEMI and self-monitoring on the main outcome changes [43].

## Methods

### Ethical Considerations

All study procedures were approved by the National University of Singapore (NUS) before participant recruitment and all participants provided informed consent (institutional review board #S-20-025). This RCT was preregistered on Open Science Framework [44]. All data were de-identified. Participants were reimbursed up to $30, 8 subject pool hour credits, or both, pro-rated based on their degree of participation.

### Study Design

In this assessor-blinded RCT, we randomized individuals into 1 of 2 arms with a parallel design and 1:1 allocation ratio. Randomization was stratified according to age and sex. We used a mixed design of 2 groups (group: MEMI vs self-monitoring) by 3 (prerandomization, postintervention, and 1MFU postrandomization) time points to evaluate the efficacy of the 14-day MEMI compared to self-monitoring on ER difficulties and self-compassion domain outcomes. Random treatment assignment to the MEMI and self-monitoring arms was the between-participant factor, whereas time points served as the within-participant factor. The trial was advertised as a “digital mindfulness intervention study” by emailing NUS students via a listserve, posting advertisements across the campus and NUS-affiliated mental health clinics, and permitting recruitment from both the student body and the broader community.

### Eligibility Criteria

Details of the study methods can be found in Multimedia Appendix 1 and in an earlier report by Zainal et al [43]. Eligible participants were required to self-report SAD with a Social Phobia Inventory (SPIN) score ≥20 [45], be aged at least 18 years, own a smartphone, and seek help for mental health issues. We recruited treatment-seeking individuals from the psychology participant pool and the local community, excluding those with self-reported suicidal ideation, mania, or psychosis. Eligible participants were recruited to this web-based trial on campus (before the COVID-19 pandemic) and on the web (during the pandemic) between September 1, 2019, and May 31, 2021.

Several reasons prompted us to choose the SPIN measure to screen for participants with probable SAD. Unlike the Social Phobia Diagnostic Questionnaire (SPDQ), the SPIN was already integrated as part of a larger battery of screening assessments in a busy psychological clinic and an undergraduate psychology research participant pool at NUS. This clinical assessment battery was based on a series of *Diagnostic and Statistical Manual (DSM), Fifth Edition*–text revised web-based assessment measures made accessible and recommended by the American Psychiatric Association [46]. Although the SPDQ has superior psychometric properties to diagnose SAD, given its structural concordance with the *DSM*, the briefer SPIN was the more pragmatic and operationally efficient choice for this study. Moreover, both the SPIN and SPDQ baseline scores were highly correlated (*r*=0.89; *P*<.001). Furthermore, all eligible participants met criteria for probable SAD at baseline using the recommended SPDQ cut-off score of ≥7.38 that had optimal sensitivity (82%), specificity (85%), a positive predictive value (83%), and a negative predictive value (83%) with a clinical diagnosis of SAD with the Anxiety Disorder Interview Schedule-IV [47,48].

### Participants

**Figure 1:**
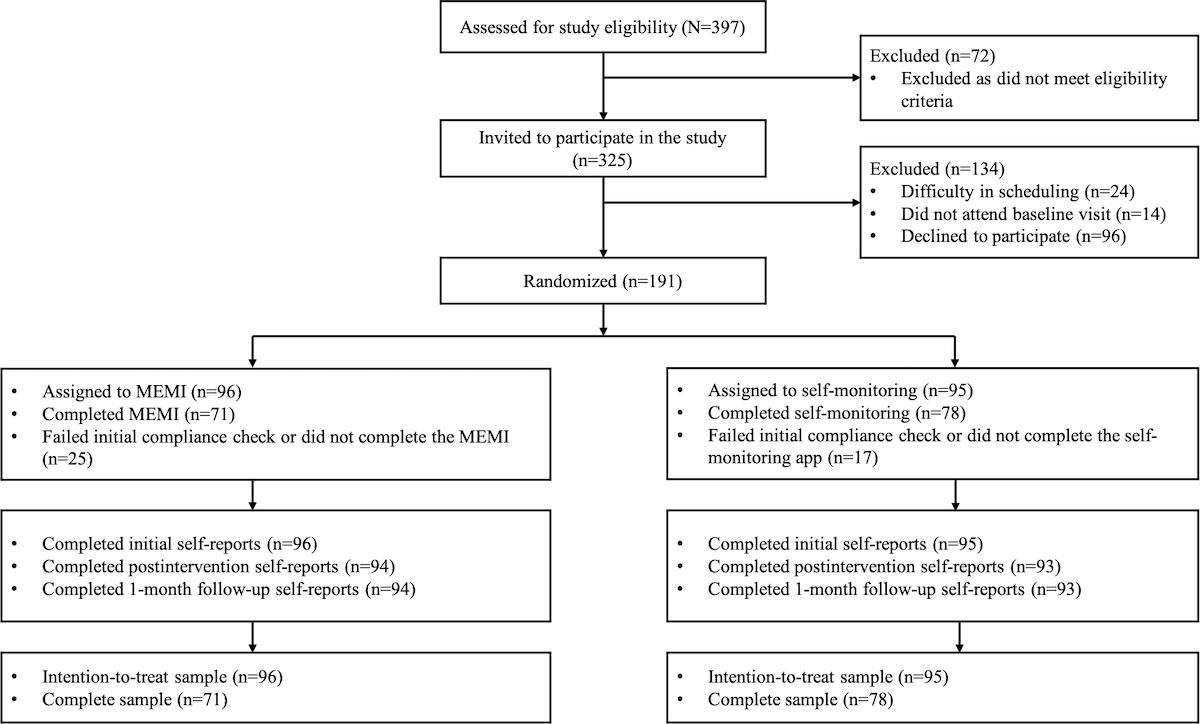
CONSORT (Consolidated Standards of Reporting Trials) diagram. MEMI: mindfulness ecological momentary intervention. SM: self-monitoring.

All participants provided voluntary informed consent. We randomized 191 participants into 2 groups: MEMI (96/191, 50.3%) and self-monitoring (95/191, 49.7%). Their average age was 21.84 (SD 3.37; range 18-53) years. Of the 191 participants, 41 (21.5%) identified as male, 149 (78%) as female, and 1 (1%) as other; 165 (86.4%) identified as Chinese and the remaining 26 (13.6%) identified as Indian, Malay, and other ethnicities; 167 (87.4%) were categorized as never married, whereas 24 (12.6%) were married, living with a partner, or in an intimate relationship but not living together; 145 (75.9%) had completed junior college as their highest level of education, whereas 46 (24.1%) held diplomas, university degrees, or graduate degrees; 139 (72.8%) were not employed, whereas 52 (27.2%) were engaged in part-time or full-time work; 178 (93.2%) were full-time students, whereas the remaining 13 (6.8%) were part-time students or nonstudents; 172 (90%) reported an annual income within the range of US $0 to US $7500, whereas 19 (9.9%) fell into higher income brackets; 11 (5.8%) had previously received a clinical diagnosis of anxiety or depressive disorder; and 10 (5.2%) were currently using psychotropic drugs (Table S1 in Multimedia Appendix 1). Eligible participants had a mean SPIN score of 35.65 (SD 13.18; range 20-67), with 31.9% (61/191) in the mild severity (score of 20-30), 31.9% (61/191) in the moderate severity (score of 31-40), 21.9% (42/191) in the severe (score of 41-50), and 14.1% (27/191) in the very severe (score of ≥51) categories [45,49,50]. Figure 1 presents the CONSORT-EHEALTH (Consolidated Standards of Reporting Trials of Electronic and Mobile Health Applications and Online Telehealth) [51] diagram (Multimedia Appendix 2).

### Procedures

The MEMI and self-monitoring arms were codeveloped by the lead author (NHZ) and senior author (MGN; both PhD-level psychologists). The study procedures were tested with various research assistants who helped provide feedback and troubleshoot technical issues and were refined from August 1, 2018, to May 31, 2019. Weekly meetings were held during this period to optimize the study procedures and reach a consensus regarding ambiguous procedural aspects. Therefore, the team did not encounter technical glitches with the Personal Analytics Companion app [52] used to deliver the MEMI and self-monitoring app during the subsequent data collection phase.

At baseline, eligible participants completed a counterbalanced series of web-based self-report measures. Subsequently, we randomly assigned participants to either the MEMI or self-monitoring arm using the Excel (Microsoft Corp) randomization function integrated into Qualtrics (Qualtrics International Inc). The mindfulness or self-monitoring video was provided toward the end of the baseline visit after the completion of all pretrial assessments. Then, participants installed the Personal Analytics Companion app [52], which contained either the MEMI or self-monitoring tool, on their smartphones, with the experimenter demonstrating its features. Assessors (also called experimenters herein) were blinded to group assignment. Participants were told that they would receive an intervention and experimenters could not know if they were randomly assigned to the MEMI or self-monitoring arm. The owner of the Personal Analytics Companion software had no role in the development of the MEMI and self-monitoring app. Participants were informed that they would receive prompts at 5 different times each day (around 9 AM, noon, 3 PM, 6 PM, and 9 PM) during the following 14-day period. These prompts could be edited within an approximately 2-hour window (ie, 8 AM-10 AM, 11 AM-1 PM, 2 PM-4 PM, 5 PM-7 PM, and 8 PM-10 PM) to fit each participant’s schedule and did not substantially differ across participants. To ensure the validity of responses, participants were instructed to provide input on their current state of depression, anxiety, and mindfulness within 2 hours of receiving the MEMI or self-monitoring prompt. These prompts guided the participants in 1-minute mindfulness or self-monitoring activities based on their assigned group. The apps did not require the training of Bachelor of Arts—level coaches and were entirely self-guided. Following the 14-day treatment phase, participants received emails prompting them to complete the self-reported clinical outcome measures at postintervention and 1MFU time points.

### RCT Arms of MEMI and Self-Monitoring

#### MEMI App

Participants assigned to the MEMI app viewed a standardized video presentation led by the principal investigator that described the evidence-based MBI protocol akin to mindfulness-based stress reduction [18,53]. This presentation familiarized MEMI participants with mindfulness, encouraging them to fully immerse themselves in their present moment and equipping them with skills encompassing open monitoring and attentiveness to present transient experiences. Proficiency in present-moment awareness may increase the richness of experiences, thereby amplifying emotional responsiveness [14]. Next, the video therapist skillfully demonstrated the paced, rhythmic diaphragmatic breathing technique and guided participants in its practice. Subsequently, the video therapist continued by imparting lessons on nonjudgmental acceptance (ie, allowing emotions to fluctuate and experiences to unfold without deliberately changing them), drawing from the mindfulness-based cognitive therapy principles [19]. These lessons fostered mindfulness-related attributes such as observation, nonreactivity, and nonjudgmental acceptance. Subsequently, each MEMI participant received a comprehensive explanation regarding the importance and benefits of consistent mindfulness practice.

The MEMI app prompted participants to practice mindfulness 5 times daily (at approximately 9 AM, noon, 3 PM, 6 PM, and 9 PM) for 14 days. During each prompt, MEMI participants received the following instructions:

Pay attention to your breathing. Breathe in a slow, steady, and rhythmic manner. Stay focused on the sensations of the air coming into your lungs and then letting it out. As you’re breathing, observe your experience as it is. Let go of judgments that do not serve you. Focus on the here and now. Attend to the small moments right now (eg, reading a chapter, having a cool glass of water), as that is where enjoyment, peace, and serenity in life happen.

Participants rated their state-level (ie, momentary) depression (“To what degree do you feel depressed right now?”), anxiety (“To what degree do you feel keyed up or on edge right now?”), and mindfulness (“To what extent are you experiencing the present moment fully?”) levels on a 9-point Likert scale (1=*not at all* to 9=*extremely*) before and after receiving these instructions. Each MEMI prompt ended with encouragement to inculcate these skills in the long term as follows:

Remember that the cultivation of mindfulness is lifelong. The goal of therapy is to be your own therapist. Practice mindfulness between the prompts and after you have completed this study.

These MEMI techniques were proposed to work for SAD in the following ways. First, *focused attention* to present-moment activities would break the habit of ruminating on the past unproductively, such as brooding over social events in self-critical ways often observed in SAD. Furthermore, this activity was designed to reduce pathological worry about the future, which is a common comorbid symptom of SAD. Second, *open monitoring* skills helped participants flexibly experience positive and negative emotions without resistance and cultivate stronger discomfort tolerance. Third, *mindful diaphragmatic breathing* might work therapeutically by inducing relaxation, improving heart rate variability, and lowering blood pressure [54]. Fourth, *acceptance* exercises might enhance the capacity to tolerate and manage emotional states, specifically addressing affect intolerance or sensitivity, an etiological and maintenance factor of SAD, and other psychopathology [55]. Unlike cognitive behavioral therapy (CBT) apps, the MEMI app does not work by continually instructing exposure therapy designed to encourage immersion in fear-inducing social situations to help persons with SAD cope with their anxiety and enhance self-efficacy in these contexts. No instructions were given to persuade participants to create a list of feared and avoided situations and to gradually and consistently approach and engage with these situations. Collectively, the MEMI app focused primarily on teaching mindfulness principles and skills.

The experimenter was available to address questions and then administered the 6-item Credibility and Expectancy Questionnaire [56]. After participants grasped the rationale and techniques of mindfulness, they set up the MEMI app on their smartphones. Furthermore, participants were provided with a copy of the MEMI rationale handout and were encouraged to engage with it consistently (Multimedia Appendix 1).

#### Self-Monitoring App

The video presentation created for self-monitoring participants commenced with the principal investigator (NHZ) explaining self-monitoring as an elevated awareness of one’s thoughts and emotions, with particular attention to one’s uncomfortable experiences. Following this, the video introduced the idea that monitoring thoughts and tracking associated distress could lead to healthier thinking. In essence, the self-monitoring video conveyed that self-monitoring (ie, focusing solely on distress) had the potential to alleviate anxiety. Self-monitoring was used in another set of studies that inspired the conceptual foundation of our self-monitoring app [57,58]. This adaptation sought to emulate the MEMI structure while intentionally excluding its presumed therapeutic elements, including acceptance, diaphragmatic breathing training, focusing on transient present moments, open monitoring, and a regimen of consistent mindfulness practices. Notably, it refrained from introducing the concept of mindfulness and abstained from guiding participants toward engaging with the present moment in ways that could influence their mood. In contrast to MEMI, the self-monitoring app encouraged participants to simply observe their thoughts and emotions and did not include emphasis on the need to accept these thoughts and feelings as they emerged. Furthermore, it did not include instructions for breathing retraining or the intention to elicit relaxation through abdominal breathing. Unlike MEMI, which promoted ongoing mindfulness practice, self-monitoring participants were not prompted to engage in self-monitoring between prompts and after the intervention. This self-monitoring approach was strategically designed to address potential credibility and expectancy effects, minimize the likelihood of regression to the mean, and mitigate the possibility of inflated effect sizes that could occur with a no-treatment or waitlist control group [59] (Multimedia Appendix 1).

Instead of receiving lengthier messages to practice mindfulness continually as with the MEMI group, self-monitoring participants received the following brief instruction 5 times daily (at about 9 AM, noon, 3 PM, 6 PM, and 9 PM) for 14 days: “Notice your thoughts and how distressing they may be.” State-level depression, anxiety, and mindfulness were measured using the same 9-point Likert scale items before and after each self-monitoring prompt. Subsequently, similar to the MEMI group, experimenters administered the 6-item Credibility and Expectancy Questionnaire after confirming self-monitoring participants’ comprehension of the rationale and self-monitoring technique. Participants received compensation through either course extra credit hours or monetary rewards.

The self-monitoring app was designed to work in the following manner. By suggesting that solely self-monitoring and focusing on distressing thought patterns might remedy anxiety, the app controlled for treatment expectancy and credibility effects across arms. Furthermore, by eliminating active therapeutic ingredients in the MEMI app (eg, focused attention on the present moment, open monitoring, diaphragmatic breathing, and nonjudgmental acceptance), the self-monitoring app functioned as a placebo comparator to maximize the odds of attaining between-arm efficacy on clinical outcomes.

### Pre- and Midintervention Measures

#### Trait ER

The Difficulties in Emotion Regulation Scale (DERS) [41] is a 36-item web-based self-report tool that evaluates difficulties in regulating emotions. Participants responded on a 5-point scale (1=*almost never, 0%-10%* to 5=*almost always, 91%-100%*). The scale provides a total score (range 36-180), where higher scores reflect more ER difficulties. In addition, 6 ER difficulty facets were evaluated via the following DERS subscales: *nonacceptance of emotions* (acceptance), *emotional awareness issues* (awareness), *lack of emotional clarity* (clarity), *goal-directed behavior difficulties* (goals), *impulse control problems* (impulsivity), and *strategy use problems* (strategy and difficulty accessing ER skills) [41]. The DERS has shown strong internal consistency, excellent 2-month retest reliability [41], and good convergent and discriminant validity [60]. The internal consistency (Cronbach α values) was excellent at .95 at both prerandomization and 7-day midintervention time points.

#### Trait Self-Compassion

Participants rated on a 5-point Likert scale (1=*almost never* to 5=*almost always*) the extent to which they identified with each statement on the Self-Compassion Scale (SCS) [8]. The SCS involves six distinct aspects assessed via the following subscales: (1) *acknowledging shared human struggles* (common humanity), (2) *social connectedness* (feeling a sense of being linked with other humans), (3) *mindfulness* (nonjudgmental awareness), (4) *nonidentification with emotions* (the absence of intense focus on negative emotions), (5) *nonjudgment toward oneself* (self-soothing tendencies during times of distress), and (6) *self-kindness* (showing warmth toward one’s imperfections) [8]. The SCS has shown strong internal consistency, predictive validity, convergent validity, discriminant validity, and retest reliability [61-63]. The Cronbach α values were good at 0.95 and 0.96 at prerandomization and 7-day midintervention time points, respectively.

#### SAD Symptoms

The 25-item SPDQ [48] assessed SAD fear and avoidance symptoms in different social situations, aligned with the *DSM Fourth Edition* criteria [64]. It has shown good retest reliability and strong internal consistency (Cronbach α=.96, .97, and .98 at prerandomization, postintervention, and 1MFU time points, respectively) [48]. Furthermore, it displayed robust convergent and discriminant validity, with high sensitivity (82%), specificity (85%) [48], and responsiveness to symptom reduction changes in clinical trials [13].

The 17-item SPIN [45] evaluated the SAD fear and avoidance symptoms in the past week, such as the fear of social embarrassment. Participants used a 5-point Likert scale (1=*not at all* to 5=*extremely*) to rate the relevance of each statement to their past week’s experiences. Prior research has shown that the SPIN exhibited acceptable convergent validity with other established assessments of SAD [50,65]. The SPIN showed strong internal consistency in this study (Cronbach α=.93, .94, and .95 at prerandomization, postintervention, and 1MFU time points, respectively). Finally, a cut-off SPIN score ≥20 yielded excellent psychometric properties, with a sensitivity of 0.85, specificity of 0.86, positive predictive value of 0.85, negative predictive value of 0.85, and correct classification rate of 85% [50], when compared to the structured interview for the *DSM Fourth Edition* [66,67]. Excellent psychometric properties with a similar cut-off have been replicated in another Asian sample [68].

#### Generalized Anxiety Symptoms

The Generalized Anxiety Disorder Questionnaire–fourth edition (GADQ-IV) [69], comprising 14 items, evaluated the symptoms of generalized anxiety disorder (GAD) through a combination of dichotomous (“yes” or “no”) and continuous responses. The continuous responses included a 9-point Likert scale for assessing the interference and distress caused by GAD symptoms. The GADQ-IV showed strong internal consistency (Cronbach α=0.93, 0.93, and 0.94 at prerandomization, postintervention, and 1MFU time points, respectively) and high retest reliability [69]. Furthermore, it exhibited strong convergent and discriminant validity, and aligned well with the structured diagnostic assessments of GAD [70,71].

#### Depression Severity

The Beck Depression Inventory–second edition (BDI-II) [72], which is a 21-item scale, assesses depression symptom severity. Participants rated the severity of each symptom based on their experiences in the past 2 weeks, using a scale of 0 to 3 to indicate increasing severity. The BDI-II showed excellent internal consistency (Cronbach α=.93, .95, .95 at prerandomization, postintervention, and 1MFU time points, respectively), high retest reliability, and strong convergent and discriminant validity [73,74].

#### Trait Repetitive Negative Thinking

The 45-item Perseverative Cognitions Questionnaire (PCQ; PCQ-45) [75] assessed persistent, repetitive negative thinking tendencies related to worrisome, obsessive, and ruminative thoughts. Participants rated the items on a 6-point Likert scale (0=*strongly disagree* to 5=*strongly agree*). The PCQ-45 included 6 domains: anticipating the worst, brooding over the past, future preparation, thoughts conflicting with the ideal self, uncontrollability, and seeking causes and meanings [75]. We calculated the total PCQ score by computing the average of all subscale scores. The scale showed high 2-week retest reliability and strong discriminant and convergent validity (Cronbach α=.96, .97, and .98 at prerandomization, postintervention, and 1MFU time points, respectively), and it demonstrated cross-cultural measurement invariance between the United States and Singapore [76].

#### Trait Mindfulness

The 39-item Five-Factor Mindfulness Questionnaire (FFMQ) [77] measured participants’ inclination to practice mindfulness across 5 domains: awareness of the consequences of actions, description, nonjudgment, nonreactivity to inner experiences, and observation. Participants rated these aspects on a 5-point Likert scale (1=*never or very rarely true* to 5=*very often or always true*). The FFMQ total score showed strong convergent validity [78], differentiation from measures of unrelated factors (eg, psychological well-being) [77], and retest reliability [79]. The Cronbach α values were .90, .91, and .93 at prerandomization, postintervention, and 1MFU time points, respectively.

### Statistical Analysis

#### Power Analysis and Missing Data Management

On the basis of a Monte Carlo power analysis [80,81], this study had 100% power to detect a substantial group × time interaction with a small effect size of Cohen *d*=0.2. We performed intent-to-treat [82] analyses with the enrolled 191 participants by incorporating data from participants who did not meet the 7-day compliance check (completing ≥80% of the app prompts in the 2-week intervention phase). Participants demonstrated strong adherence, with 78% (149/191) responding to at least 80% (56/70) of the MEMI and self-monitoring prompts. A total of 2.03% of the data from the pre-1MFU time points were missing. To address this, we applied multiple imputation using the recommended predictive mean matching algorithm by pooling data from 100 imputed data sets, each with 10 iterations [83].

#### Hierarchical Linear Modeling

To test hypothesis 1, we used hierarchical linear modeling [84], also known as multilevel modeling, to account for data nonindependence resulting from the nesting of repeated observations (level 1) within participants (level 2). For each hierarchical linear modeling, group (intervention), time, and group × time interaction were fixed-effect predictors of improvement in the prerandomization to midintervention ER and self-compassion domains, and the intercept (ie, time-coded as 0 for preintervention and 1 for midintervention time points) was the single random-effect predictor (permitting participants to vary in their average-outcome values). We used fitted models to determine the estimated mean scores at each time point.

#### Parallel Structural Equation Modeling Mediation Analyses

To test hypothesis 2, we conducted parallel mediation analyses using the *lavaan* structural equation modeling R package (R Foundation for Statistical Computing) [85] to assess whether pretrial to midintervention ER and self-compassion domains mediated group effects on the 6 clinical outcomes from pre-post and pre-1MFU time points. Similar to the primary RCT study, the clinical outcomes examined were the alleviation of pre-post and pre-1MFU SAD symptoms, generalized anxiety symptoms, depression severity, repetitive negative thinking, and trait mindfulness. Although there were no significant between-group effects from pre-post and pre-1MFU time points [43], we conducted mediation tests because significant indirect effects can exist without significant between-group total or direct effects [86]. Furthermore, our primary efficacy paper still showed significant within-group effects on these 5 clinical outcomes from pre-post and pre-1MFU time points [43]. Moreover, as recommended, our mediation tests aligned with theoretical considerations [87] and statistical recommendations to measure mediators at the midpoint of the intervention. In order to establish them as potential mechanisms of change, mediators should temporally precede the outcome measures [88]. The associations between groups, pretrial to midintervention mediators, and pre-post or pre-1MFU outcomes can be described using 4 regression coefficients (or paths) [89]: the group effect on the pretrial to midintervention mediator (path *a*), the effect of the pretrial to midintervention mediator on pre-post or pre-1MFU outcome (path *b*), the total effect (path *c*; the combination of the *a* and *b* paths), and the direct effect (path *c*’; the group effect on the outcome, irrespective of the mediator). The product of paths *a* and *b* signifies the mediated (or indirect) effect, that is, the focal estimate when examining mediation. We assessed the significance of the mediated effect via a nonparametric bootstrap method, generating 2000 random samples to determine point estimates, SEs, and bias-corrected bootstrap 95% CIs for each indirect effect. The mediated effect was considered significant if the CI bounds (both upper and lower) did not include 0. To safeguard against type I errors, we conducted post hoc comparisons for statistically significant effects using the Simes Bonferroni correction method [90]. Cohen *d* effect sizes and their 95% CIs were calculated to ease the interpretation of parameter estimates for both study hypotheses, such that Cohen *d* values of 0.2, 0.5, and 0.8 denote small, moderate, and large effects, respectively [91].

## Results

Table S2 in Multimedia Appendix 1 displays descriptive statistics of the trait ER and self-compassion domain scores for participants in the MEMI and self-monitoring groups.

### Between- and Within-Group Effects of Brief MEMI Versus Self-Monitoring on ER Domains

Significant between-group effects occurred from pretrial to midintervention time points on DERS domains, including lack of emotional clarity (Cohen *d*=0.16, 95% CI 0.02-0.31; *P=*.03) and difficulties engaging in goal-directed behavior (Cohen *d*=−0.24, 95% CI −0.39 to −0.10; *P=*.001; Tables 1 and 2). Regarding within-group effects, reductions in these difficulties were significantly stronger for the MEMI than self-monitoring groups from pretrial to midintervention time points (lack of emotional clarity: Cohen *d*=−0.39, 95% CI −0.54 to −0.25; *P<*.001 vs Cohen *d*=−0.21, 95% CI −0.35 to −0.06; *P=*.004 and goal-directed behavior difficulties: Cohen *d*=−0.73, 95% CI −0.88 to −0.58; *P<*.001 vs Cohen *d*=−0.29, 95% CI −0.44 to −0.15; *P<*.001). No significant between-group effects emerged from pretrial to midintervention time points on emotional awareness issues (Cohen *d*=0.11, 95% CI −0.03 to 0.26; *P=*.12), impulse control issues (Cohen *d*=0.11, 95% CI −0.03 to 0.26; *P=*.12), nonacceptance of emotions (Cohen *d*=−0.07, 95% CI −0.21 to 0.08; *P=*.36), and strategy use problems (Cohen *d*=−0.12, 95% CI −0.27 to 0.02; *P=*.10). Regarding within-group effects, both the MEMI and self-monitoring app significantly reduced impulse control issues (MEMI: Cohen *d*=−0.32, 95% CI −0.47 to −0.18; *P=*.006 and self-monitoring: Cohen *d*=−0.26, 95% CI −0.40 to −0.11; *P<*.001), nonacceptance of emotions (MEMI: Cohen *d*=−0.32, 95% CI −0.47 to −0.18; *P<*.001; self-monitoring: Cohen *d*=−0.24, 95% CI −0.38 to −0.09; *P=*.001), and strategy use problems (MEMI: Cohen *d*=−0.40, 95% CI −0.54 to −0.25; *P<*.001; self-monitoring: Cohen *d*=−0.21, 95% CI −0.35 to −0.06; *P=*.004) from pretrial to midintervention time points. A significant reduction in emotional awareness issues from pretrial to midintervention time points was observed in the self-monitoring group (Cohen *d*=−0.73, 95% CI −0.88 to −0.58; *P<*.001) but not in the MEMI group (Cohen *d*=−0.29, 95% CI −0.44 to 0.02; *P=*.07).

**Table 1:** Between-intervention hierarchical linear modeling of the mindfulness ecological momentary intervention versus self-monitoring app predicting emotion regulation and self-compassion domains.

Domains				β		*t* test (*df*)		*P* value		Cohen *d* (95% CI)
**DERS^a^ domains**										
	**DERS emotional awareness issues**									
		Intercept	16.66		39.37 (953)		<.001		2.85 (2.64 to 3.05)	
		Group	−.58		−0.97 (189)		.34		−0.07 (−0.21 to 0.07)	
		Prerandomization to midintervention time points	−.91		−3.97 (953)		<.001		−0.29 (−0.43 to −0.14)	
		Group × pretrial to midintervention time points	.50		1.54 (953)		.12		0.11 (−0.03 to 0.26)	
	**DERS lack of emotional clarity**									
		Intercept	13.02		38.69 (953)		<.001		2.80 (2.60 to 3.00)	
		Group	−0.19		−0.41 (189)		.68		−0.03 (−0.17 to 0.12)	
		Prerandomization to midintervention time points	−1.31		−7.33 (953)		<.001		−0.53 (−0.68 to −0.38)	
		Group × pretrial to midintervention time points	.56		2.21 (953)		.027		0.16 (0.02 to 0.31)	
	**DERS goal-directed behavior difficulties**									
		Intercept	17.22		43.55 (953)		<.001		3.15 (2.93 to 3.37)	
		Group	.40		0.71 (189)		.48		0.05 (−0.09 to 0.20)	
		Prerandomization to midintervention time points	−1.02		−4.40 (953)		<.001		−0.32 (−0.46 to −0.17)	
		Group × pretrial to midintervention time points	−1.11		−3.39 (953)		.001		−0.24 (−0.39 to −0.10)	
	**DERS impulse control issues**									
		Intercept	16.66		31.84 (953)		<.001		2.85 (2.64 to 3.05)	
		Group	−.58		0.12 (189)		.34		−0.07 (−0.21 to 0.07)	
		Prerandomization to midintervention time points	−.91		−3.30 (953)		<.001		−0.29 (−0.43 to −0.14)	
		Group × pretrial to midintervention time points	.50		0.29 (953)		.12		0.11 (−0.03 to 0.26)	
	**DERS nonacceptance of emotions**									
		Intercept	15.74		32.08 (953)		<.001		2.32 (2.13 to 2.51)	
		Group	−.30		−0.43 (189)		.67		−0.03 (−0.18 to 0.11)	
		Prerandomization to midintervention time points	−1.01		−3.20 (953)		.001		−0.23 (−0.38 to −0.09)	
		Group × pretrial to midintervention time points	−.41		−0.92 (953)		.36		−0.07 (−0.21 to 0.08)	
	**DERS strategy use problems**									
		Intercept	21.23		35.21 (953)		<.001		2.55 (2.35 to 2.74)	
		Group	−.36		−0.42 (189)		.68		−0.03 (−0.17 to 0.11)	
		Prerandomization to midintervention time points	−.93		−2.94 (953)		.003		−0.21 (−0.36 to −0.07)	
		Group × pretrial to midintervention time points	−.75		−1.67 (953)		.10		−0.12 (−0.27 to 0.02)	
**SCS^b^ domains**										
	**SCS acknowledging human struggles**									
		Intercept	12.20		34.92 (953)		<.001		2.53 (2.33 to 2.72)	
		Group	−.24		−0.49 (189)		.63		−0.04 (−0.18 to 0.11)	
		Prerandomization to midintervention time points	−.61		−3.48 (953)		.001		−0.25 (−0.40 to −0.11)	
		Group × pretrial to midintervention time points	.88		3.56 (953)		<.001		0.26 (0.11 to 0.40)	
	**SCS social connectedness**									
		Intercept	10.25		31.05 (953)		<.001		2.25 (2.06 to 2.43)	
		Group	.15		0.31 (189)		.75		0.02 (−0.12 to 0.17)	
		Prerandomization to midintervention time points	.41		2.39 (953)		.02		0.17 (0.03 to 0.32)	
		Group × pretrial to midintervention time points	.73		3.00 (953)		.003		0.22 (0.07 to 0.36)	
	**SCS mindfulness**									
		Intercept	12.45		44.87 (953)		<.001		3.25 (3.03 to 3.47)	
		Group	−.21		−0.53 (189)		.60		−0.04 (−0.18 to 0.11)	
		Prerandomization to midintervention time points	.04		0.29 (953)		.78		0.02 (−0.12 to 0.17)	
		Group × pretrial to midintervention time points	.01		0.04 (953)		.97		0.00 (−0.14 to 0.15)	
	**SCS nonidentification with emotions**									
		Intercept	10.05		34.19 (953)		<.001		2.47 (2.28 to 2.67)	
		Group	−0.25		−0.60 (189)		.55		−0.04 (−0.19 to 0.10)	
		Prerandomization to midintervention time points	1.02		6.18 (953)		<.001		0.45 (0.30 to 0.59)	
		Group × pretrial to midintervention time points	.50		2.15 (953)		.03		0.16 (0.01 to 0.30)	
	**SCS nonjudgment toward oneself**									
		Intercept	12.74		33.37 (953)		<.001		2.41 (2.22 to 2.60)	
		Group	.18		0.33 (189)		.74		0.02 (−0.12 to 0.17)	
		Prerandomization to midintervention time points	1.29		6.66 (953)		<.001		0.48 (0.34 to 0.63)	
		Group × pretrial to midintervention time points	.46		1.66 (953)		.10		0.12 (−0.02 to 0.27)	
	**SCS self-kindness**									
		Intercept	14.12		37.95 (953)		<.001		2.75 (2.54 to 2.95)	
		Group	.28		0.53 (189)		.60		0.04 (−0.11 to 0.18)	
		Prerandomization to midintervention time points	.24		1.33 (953)		.18		0.10 (−0.05 to 0.24)	
		Group × pretrial to midintervention time points	.67		2.61 (953)		.009		0.19 (0.04 to 0.33)	

^a^DERS: Difficulties in Emotion Regulation Scale.

^b^SCS: Self-Compassion Scale.

**Table 2:** Within-intervention hierarchical linear modeling of MEMI^a^ and self-monitoring app predicting emotion regulation and self-compassion domains.

Domains				β		*t* test (*df*)		*P* value		Cohen *d* (95% CI)
**DERS^b^ domains**										
	**DERS emotional awareness issues**									
		Intercept (MEMI)	16.08		36.33 (479)		<.001		2.64 (2.45 to 2.84)	
		Time (MEMI)	−.41		–1.79 (479)		.07		−0.13 (−0.27 to 0.02)	
		Intercept (self-monitoring)	16.66		41.46 (474)		<.001		3.01 (2.80 to 3.23)	
		Time (self-monitoring)	−.91		–4.00 (474)		<.001		−0.29 (−0.43 to −0.14)	
	**DERS lack of emotional clarity**									
		Intercept (MEMI)	20.87		35.74 (479)		<.001		2.60 (2.41 to 2.80)	
		Time (MEMI)	−1.68		−5.47 (479)		<.001		−0.39 (−0.54 to −0.25)	
		Intercept (self-monitoring)	21.23		34.31 (474)		<.001		2.48 (2.29 to 2.68)	
		Time (self-monitoring)	−.93		−2.87 (474)		.004		−0.21 (−0.35 to −0.06)	
	**DERS goal-directed behavior difficulties**									
		Intercept (MEMI)	17.61		46.44 (479)		<.001		3.36 (3.14 to 3.59)	
		Time (MEMI)	−2.13		−10.11 (479)		<.001		−0.73 (−0.88 to −0.58)	
		Intercept (self-monitoring)	17.22		42.07 (474)		<.001		3.04 (2.83 to 3.26)	
		Time (self-monitoring)	−1.02		−4.07 (474)		<.001		−0.29 (−0.44 to −0.15)	
	**DERS impulse control issues**									
		Intercept (MEMI)	14.94		32.73 (479)		<.001		2.28 (2.09 to 2.46)	
		Time (MEMI)	−.67		−2.73 (479)		.006		−0.32 (−0.47 to −0.18)	
		Intercept (self-monitoring)	14.87		31.26 (474)		<.001		2.26 (2.08 to 2.45)	
		Time (self-monitoring)	−.76		−3.56 (474)		<.001		−0.26 (−0.40 to −0.11)	
	**DERS nonacceptance of emotions**									
		Intercept (MEMI)	15.44		31.45 (479)		<.001		2.28 (2.09 to 2.46)	
		Time (MEMI)	−1.42		−4.45 (479)		<.001		−0.32 (−0.47 to −0.18)	
		Intercept (self-monitoring)	15.74		32.26 (474)		<.001		2.33 (2.15 to 2.52)	
		Time (self-monitoring)	−1.01		−3.26 (474)		.001		−0.24 (−0.38 to −0.09)	
	**DERS strategy use problems**									
		Intercept (MEMI)	20.87		35.74 (479)		<.001		2.59 (2.39 to 2.78)	
		Time (MEMI)	−1.68		−5.47 (479)		<.001		−0.40 (−0.54 to −0.25)	
		Intercept (self-monitoring)	21.23		34.31 (474)		<.001		2.48 (2.29 to 2.68)	
		Time (self-monitoring)	−.93		−2.87 (474)		.004		−0.21 (−0.35 to −0.06)	
**SCS^c^ domains**										
	**SCS acknowledging human struggles**									
		Intercept (MEMI)	11.96		33.83 (479)		<.001		2.45 (2.26 to 2.64)	
		Time (MEMI)	.27		1.74 (479)		.08		0.13 (−0.02 to 0.27)	
		Intercept (self-monitoring)	12.20		35.52 (474)		<.001		2.57 (2.37 to 2.77)	
		Time (self-monitoring)	−.61		−3.18 (474)		.002		−0.23 (−0.38 to −0.08)	
	**SCS social connectedness**									
		Intercept (MEMI)	10.40		29.86 (479)		<.001		2.16 (1.98 to 2.34)	
		Time (MEMI)	1.14		6.20 (479)		<.001		0.45 (0.30 to 0.60)	
		Intercept (self-monitoring)	10.25		33.21 (474)		<.001		2.40 (2.21 to 2.59)	
		Time (self-monitoring)	.41		2.59 (474)		.01		0.19 (0.04 to 0.33)	
	**SCS mindfulness**									
		Intercept (MEMI)	12.25		42.63 (479)		<.001		3.08 (2.87 to 3.30)	
		Time (MEMI)	.05		0.35 (479)		.73		0.03 (−0.12 to 0.17)	
		Intercept (self-monitoring)	12.45		46.91 (474)		<.001		3.39 (3.17 to 3.62)	
		Time (self-monitoring)	.04		0.29 (474)		.77		0.02 (−0.12 to 0.17)	
	**SCS nonidentification with emotions**									
		Intercept (MEMI)	9.80		32.35 (479)		<.001		2.34 (2.15 to 2.53)	
		Time (MEMI)	1.52		8.74 (479)		<.001		0.63 (0.48 to 0.78)	
		Intercept (self-monitoring)	10.05		35.53 (474)		<.001		2.57 (2.38 to 2.77)	
		Time (self-monitoring)	1.02		6.60 (474)		<.001		0.48 (0.33 to 0.62)	
	**SCS self-judgment**									
		Intercept (MEMI)	12.92		34.08 (479)		<.001		2.47 (2.27 to 2.66)	
		Time (MEMI)	1.75		8.39 (479)		<.001		0.61 (0.46 to 0.76)	
		Intercept (self-monitoring)	12.74		33.28 (474)		<.001		2.41 (2.22 to 2.6)	
		Time (self-monitoring)	1.29		7.30 (474)		<.001		0.53 (0.38 to 0.68)	
	**SCS self-kindness**									
		Intercept (MEMI)	14.40		35.81 (479)		<.001		2.59 (2.39 to 2.79)	
		Time (MEMI)	.91		4.91 (479)		<.001		0.36 (0.21 to 0.50)	
		Intercept (self-monitoring)	14.12		41.90 (474)		<.001		3.03 (2.82 to 3.24)	
		Time (self-monitoring)	.24		1.37 (474)		.17		0.10 (−0.05 to 0.24)	

^a^MEMI: mindfulness ecological momentary intervention.

^b^DERS: Difficulties in Emotion Regulation Scale.

^c^SCS: Self-Compassion Scale.

### Between- and Within-Group Effects of Brief MEMI Versus Self-Monitoring on Self-Compassion Domains

Significant between-group effects occurred from pretrial to midintervention time points on SCS domains, including acknowledging shared human struggles (Cohen *d*=0.26, 95% CI 0.11-0.40; *P<*.001), social connectedness (Cohen *d*=0.19, 95% CI 0.04-0.33; *P=*.01), nonidentification with emotions (Cohen *d*=0.16, 95% CI 0.01-0.30; *P=*.03), and self-kindness (Cohen *d*=0.19, 95% CI 0.04-0.33; *P=*.009; Tables S3 and S4 in Multimedia Appendix 1). Regarding within-group effects, increases in these self-compassion domains were significantly larger for the MEMI than self-monitoring groups from pretrial to midintervention time points (acknowledging shared human struggles: Cohen *d*=0.13, 95% CI −0.02 to 0.27; *P=*.08 vs Cohen *d*=−0.23, 95% CI −0.38 to −0.08; *P=*.002); social connectedness: Cohen *d*=0.45, 95% CI 0.30-0.60; *P<*.001 vs Cohen *d*=0.19, 95% CI 0.04-0.33; *P=*.01; identification with emotions: Cohen *d*=0.63, 95% CI 0.48-0.78; *P<*.001 vs Cohen *d*=0.48, 95% CI 0.33-0.62; *P<*.001; and self-kindness: Cohen *d*=0.36, 95% CI 0.21-0.50; *P<*.001 vs Cohen *d*=0.10, 95% CI −0.05 to 0.24; *P=*.17). No significant between-group effects emerged from pretrial to midintervention time points on mindfulness (Cohen *d*<0.01, 95% CI −0.14 to 0.15; *P=*.97) and nonjudgment toward oneself (Cohen *d*=0.12, 95% CI −0.02 to 0.27; *P=*.10) domains. Neither the MEMI (Cohen *d*=0.03, 95% CI −0.12 to 0.17; *P=*.73) nor self-monitoring app (Cohen *d*=0.02, 95% CI −0.12 to 0.17; *P=*.77) significantly changed mindfulness from pretrial to midintervention time points. However, both the MEMI (Cohen *d*=0.61, 95% CI 0.46-0.76; *P<*.001) and self-monitoring app (Cohen *d*=0.53, 95% CI 0.38-0.68; *P<*.001) significantly increased nonjudgment toward oneself from pretrial to midintervention time points.

### Testing ER and Self-Compassion as Mediators of Outcomes From Pre-Post Time Points

Neither ER nor self-compassion domains significantly mediated the effect of the treatment (brief MEMI vs self-monitoring) on pre- and postintervention SAD symptoms indexed by SPDQ and SPIN scores, generalized anxiety symptoms (GADQ-IV score), depression severity (BDI-II score), trait repetitive negative thinking (PCQ score), and trait mindfulness (FFMQ score; Table S3 in Multimedia Appendix 1). Therefore, hypothesis 2a was not supported.

### Testing ER and Self-Compassion as Mediators of Outcomes From Pre-1MFU Time Points

Neither ER nor self-compassion domains significantly mediated the effect of the treatment (brief MEMI vs self-monitoring) on pre-1MFU SAD symptoms indexed by SPDQ and SPIN scores, generalized anxiety symptoms (GADQ-IV score), depression severity (BDI-II score), trait repetitive negative thinking (PCQ score), and trait mindfulness (FFMQ score; Table S4 in Multimedia Appendix 1). Therefore, hypothesis 2b was not supported. In a sensitivity analysis of single-mediator models, we found that mediation effects were still not statistically significant when the overall scores of DERS or SCS were tested as a single mediator for all 6 clinical outcomes.

## Discussion

### Comparison With Prior Work

This study determined the efficacy of a 14-day, fully self-guided, mobile MEMI on ER difficulties and self-compassion domains from pretrial to midintervention time points and whether such changes mediated 6 clinical outcomes from pre-post and pre-1MFU time points. Extending a prior report of primary outcomes [43], this RCT determined which domains of ER difficulties and self-compassion a brief MEMI could impact from pretrial to midintervention time points. Encouragingly, the results showed that, similar to applied relaxation and self-compassion training [92], brief, fully self-guided, mobile MEMIs efficaciously increased specific self-compassion domains. The MEMI enhanced acknowledging shared human struggles (common humanity), social connectedness (sense of being linked with other people), nonidentification with emotions (not overassociating with one’s feelings), and self-kindness domains from pretrial to midintervention time points. Therefore, the MEMI was superior to self-monitoring in increasing the ER domains linked with goal-directed behavior pursuit and emotional clarity.

Why was the brief MEMI more efficacious than self-monitoring in enhancing social connectedness, nonidentification with emotions, self-kindness, acknowledging shared human struggles, reducing goal-directed behavior difficulties, and lack of emotional clarity from pretrial to midintervention time points? Consistent with the mindfulness-to-meaning theory [93], the MEMI might have helped cultivate nonjudgmental awareness of one’s and others’ experiences, steering clear of both rumination and the suppression of distressing emotions typical of SAD. By teaching the regulation of physiological anxiety and the practice of self-kindness during moments of confusion or insecurity, the brief MEMI might offer greater benefits than self-monitoring in managing processes related to SAD. This approach might encourage constructive engagement with experiences instead of getting stuck in the vicious cycle of avoidance and missing out on positive interactions that could disconfirm feared social outcomes and offer feelings of self-efficacy across diverse interpersonal contexts [94,95]. Furthermore, the emphasis on acceptance and constructive awareness by the MEMI instead of self-monitoring might have contributed to these findings. Prior dismantling studies of MBIs that evidenced stronger comparative efficacy of acceptance and monitoring over monitoring alone on clinically relevant outcomes have alluded to this possibility [96].

Moreover, it is noteworthy that both brief MEMI and self-monitoring reduced ER difficulties linked to impulse control issues, nonacceptance of emotional responses, strategy use problems, and nonjudgment toward oneself from pretrial to midintervention time points. These null between-group effects could be viewed as a comparison between awareness alone and awareness combined with acceptance, where acceptance did not contribute anything additional to generate differential comparative efficacy on these outcomes. Perhaps learning via both the MEMI and self-monitoring to calmly acknowledge current thoughts, emotions, and bodily sensations without judgment, regardless of intensity or discomfort, fostered the ability to endure distress and engage with experiences rather than avoid them [97]. Furthermore, these processes might have motivated actions and curbed the inclination to engage in self-sabotaging behaviors, including self-criticism and impulsive actions [98,99]. In addition, these alterations may have been due to placebo and related factors rather than the influence of “active ingredients” [100]. Without the MEMI showing superior efficacy over self-monitoring on these outcomes, we cannot determine whether the improvements exceeded the effects of the passage of time, expectancy effects, and related factors. Future RCTs could empirically evaluate the validity of these conjectures.

Notably, self-monitoring, but not the MEMI, led to an increased emotional awareness. This surprising finding could be attributed to repeated instructions by self-monitoring prompts to attend to thoughts and feelings with explicit instructions to notice how distressing they might be. Another explanation might be that self-monitoring could have enhanced emotional self-awareness and related mental health outcomes, such as psychological well-being [101].

Another counterintuitive finding was the absence of between- and within-group effectiveness of the MEMI on the mindfulness domain of the SCS from pretrial to midintervention time points. A potential account for these nonsignificant outcomes was that more frequent, intense, and longer-duration mindfulness practices were necessary. Consistent with this inference, a 2-week MEMI positively affected trait mindfulness from pre-1MFU but not from pre- to postintervention time points [102]. Alternatively, the mindfulness measure used in this study might lack sensitivity to detect a change in mindfulness from pretrial to midintervention time points.

In addition, contrary to our expectations, no examined pretrial to midintervention ER or self-compassion domains were mediators of the effect of MEMI on the pre-post and pre-1MFU SAD symptoms, generalized anxiety symptoms, depression severity, repetitive negative thinking, and trait mindfulness. Other studies on brief MEMIs similarly found that ER and self-compassion did not consistently mediate treatment effects on mental health outcomes in various populations, such as distressed college students [103], health care workers [104], and other nonclinical samples [105]. These nonsignificant mediation effects might suggest that ER and self-compassion domains were not the change mechanisms of brief, fully self-guided, mobile MEMIs, as previously suggested [31,32]. On the basis of prior mechanism-focused trials that delivered higher-intensity MBIs [106-110], longer-duration and more rigorous MEMIs may be necessary to observe ER and self-compassion domains as mediators of the effect of brief MEMI on various clinical outcomes in SAD. Higher-intensity MBIs may nurture stronger consolidation of mindfulness and related ER skills over long durations and across various contexts by modifying habitual responses to social and associated stressors in SAD [111]. Furthermore, it is possible that the ER and self-compassion measures used in this study were not sensitive enough to detect any existing true mediation effects. Alternatively, based on emerging evidence, other constructs, such as increased acceptance-attention, nonreactivity [112], and cognitive reappraisal [113], should be examined as mediators or proxy change mechanisms of brief MEMI on diverse SAD outcomes. Brief MEMIs could potentially enhance alternative mechanisms that boost mental well-being, coping skills, and related factors in individuals with SAD. These testable ideas await experimental evaluation.

### Limitations and Strengths

This study had some limitations. First, all putative mediators and clinical outcome measures relied on web-based self-reports. Future RCTs should incorporate diverse measures of related constructs and evaluate crucial yet understudied factors believed to drive change, such as attentional bias toward threats [114,115], executive functioning [102,116,117], and physiological anxiety indices [118,119]. Second, we did not evaluate the sustainability of mindfulness skill use after the 2-week intervention duration. Third, we measured difficulties in ER and self-compassion domains at prerandomization and midintervention time points but not at posttreatment and 1MFU time points. This approach enabled us to evaluate the initial mechanism conditions and changes during the active treatment phase and test whether such conditions led to pre-1MFU changes in clinical outcomes [120]. Future investigations should delve into whether persistent mindfulness engagement, even without recurring MEMI instructions, can predict efficacy and change mechanisms at follow-up evaluations [121]. Fourth, the level of intensity in the brief MEMI may have been insufficient to produce substantial improvement in clinical outcomes via putative mediators. Finally, we used a clinical cut-off score on a self-report measure to determine a SAD diagnosis, instead of relying on a diagnostic interview.

Despite these limitations, this study had notable strengths, including a robust RCT design featuring an active comparator and high adherence rates, an uncommon achievement in the digital mental health intervention field, which often faces challenges such as high dropout and low use [122]. Furthermore, participants exhibited good compliance, with 78% (149/191) engaging in at least 80% of the MEMI and self-monitoring prompts. In addition, these measures have a well-established history of use and psychometric validation in previous trials [13,102,123,124]. Another strength of our study was the recruitment of a sample from Singapore, a Southeast Asian country. This method strengthened the potential for cross-cultural applicability, addressing a vital lacuna within the field of clinical psychology, which has traditionally concentrated on Western settings [76,125].

### Conclusions

If replicated, our study has several clinical implications. Our observed advantages of a brief MEMI versus self-monitoring in decreasing isolation (sense of social disconnection) and overidentification (excessive attention to negative emotions) and increasing self-kindness (tenderness toward flaws in oneself), goal-directed behaviors, and emotional clarity in people with SAD indicated that established first-line treatments, such as CBT, could benefit from integrating self-compassion and mindfulness strategies. The finding that brief MEMI was not significantly different than self-monitoring in decreasing impulse control difficulties, nonacceptance of emotional responses, limited access to ER strategies, and self-judgment (excessive self-criticism) implies that the benefits of self-monitoring alone could be highlighted in the treatment to promote self-efficacy before moving on to graded exposure therapy and other CBT components for SAD. Higher-intensity brief, fully self-guided mobile MEMIs are likely necessary to identify which ER and self-compassion domains are change mechanisms [126]. In addition to identifying change mechanisms and establishing efficacy, it will be essential to investigate which individuals with SAD will derive the greatest benefit from the brief MEMI.

## Appendix

Multimedia Appendix 1 Study methodology details.

Multimedia Appendix 2 CONSORT-EHEALTH (Consolidated Standards of Reporting Trials of Electronic and Mobile Health Applications and Online Telehealth) diagram.

## Abbreviations

1MFU: 1-month follow-up
BDI-II: Beck Depression Inventory–second edition
CBT: cognitive behavioral therapy
CONSORT-EHEALTH: Consolidated Standards of Reporting Trials of Electronic and Mobile Health Applications and Online Telehealth
DERS: Difficulties in Emotion Regulation Scale
DSM: Diagnostic and Statistical Manual
ER: emotion regulation
FFMQ: Five-Factor Mindfulness Questionnaire
GAD: generalized anxiety disorder
GADQ-IV: Generalized Anxiety Disorder Questionnaire–fourth edition
MBI: mindfulness-based intervention
MEMI: mindfulness ecological momentary intervention
NUS: National University of Singapore
OSF: Open Science Framework
PCQ: Perseverative Cognitions Questionnaire
RCT: randomized controlled trial
SAD: social anxiety disorder
SCS: Self-Compassion Scale
SPDQ: Social Phobia Diagnostic Questionnaire
SPIN: Social Phobia Inventory
