# Supplementary material for: Testing the Efficacy of a Brief, Self-Guided Mindfulness Ecological Momentary Intervention on Emotion Regulation and Self-Compassion in Social Anxiety Disorder: Randomized Controlled Trial
Source: JMIR Ment Health. 2024 Apr 19;11:e53712. doi: 10.2196/53712 (PMC11069101; doi:10.2196/53712)

**Multimedia Appendix 1**

Table S1

*Sociodemographic characteristics of participants (N = 191)*

|  | *M* | (*SD*) |
| --- | --- | --- |
| Age | 21.84 | (3.37) |
|  | *N* | (%) |
| Gender |  |  |
| Male | 41 | (21) |
| Female | 149 | (78) |
| Other | 1 | (1) |
| Ethnicity |  |  |
| Chinese | 165 | (86) |
| Malays | 4 | (2) |
| Indians | 12 | (6) |
| Others | 10 | (6) |
| Marital status |  |  |
| Married with spouse | 1 | (0.5) |
| Living with partner | 1 | (0.5) |
| In an intimate relationship but not living together | 22 | (11) |
| Never married | 167 | (87) |
| Education |  |  |
| Junior College | 145 | (76) |
| Diploma | 12 | (66) |
| University Degree | 27 | (14) |
| Graduate Degree | 7 | (4) |
| Employment status |  |  |
| Full-time | 12 | (6) |
| Part-time | 40 | (21) |
| Not employed | 139 | (73) |
| Student status |  |  |
| Full-time | 178 | (93) |
| Part-time | 5 | (3) |
| Not a student | 8 | (4) |
| Annual salary |  |  |
| $0 - $10,000 | 172 | (90) |
| $10,001 - $20,000 | 3 | (2) |
| $20,001 - $40,000 | 7 | (3) |
| $40,001 - $65,000 | 6 | (3) |
| $65,001 - $100,000 | 3 | (2) |
| Psychotropic medication |  |  |
| No | 181 | (95) |
| Yes | 10 | (5) |

*N*, total sample size; *M*, observed mean; *SD*, standard deviation of *M*; Min, minimum; Max, maximum; *SE*, standard error of skewness.

Table S2

*Descriptive statistics of all emotion regulation and self-compassion domains at all pre-randomization and mid-intervention time points in the 14-day mindfulness ecological momentary intervention (MEMI) and self-monitoring app (SM)*

|  | *n* | *M* | (*SD*) | *Median* | *Skewness* | *Kurtosis* | (*SE*) |
| --- | --- | --- | --- | --- | --- | --- | --- |
| *Pre-randomization (baseline) for MEMI* | | | | | | | |
| DERS Awareness | 96 | 16.18 | (4.98) | 16.0 | 0.41 | -0.24 | (0.51) |
| DERS Clarity | 96 | 12.85 | (4.04) | 12.0 | 0.55 | -0.50 | (0.41) |
| DERS Goals | 96 | 17.69 | (3.93) | 19.0 | -0.54 | -0.30 | (0.40) |
| DERS Impulsivity | 96 | 14.96 | (5.05) | 15.0 | 0.23 | -0.73 | (0.52) |
| DERS Nonacceptance | 96 | 15.50 | (5.78) | 14.0 | 0.61 | -0.30 | (0.59) |
| DERS Strategy | 96 | 20.88 | (6.18) | 20.0 | 0.42 | -0.31 | (0.63) |
| SCS Common humanity | 96 | 11.95 | (3.81) | 12.0 | -0.11 | -0.70 | (0.39) |
| SCS Isolation | 96 | 10.38 | (3.77) | 10.0 | 0.31 | -0.53 | (0.39) |
| SCS Mindfulness | 96 | 12.25 | (3.09) | 12.0 | -0.35 | 0.09 | (0.32) |
| SCS Overidentification | 96 | 9.80 | (2.98) | 10.0 | 0.15 | -0.14 | (0.30) |
| SCS Self-judgment | 96 | 12.92 | (3.90) | 13.0 | 0.21 | -0.56 | (0.40) |
| SCS Self-kindness | 96 | 14.42 | (4.17) | 15.0 | -0.18 | 0.09 | (0.43) |
| *Pre-randomization (baseline) for SM* | | | | | | | |
| DERS Awareness | 95 | 16.68 | (4.58) | 16.0 | 0.24 | -0.47 | (0.47) |
| DERS Clarity | 95 | 13.01 | (3.92) | 13.0 | 0.41 | -0.05 | (0.40) |
| DERS Goals | 95 | 17.22 | (4.71) | 17.0 | -0.26 | -0.91 | (0.48) |
| DERS Impulsivity | 95 | 14.82 | (5.31) | 14.0 | 0.19 | -1.04 | (0.54) |
| DERS Nonacceptance | 95 | 15.75 | (5.75) | 16.0 | 0.16 | -0.87 | (0.59) |
| DERS Strategy | 95 | 21.20 | (6.94) | 21.0 | 0.26 | -0.79 | (0.71) |
| SCS Common humanity | 95 | 12.20 | (3.60) | 12.0 | 0.05 | -0.58 | (0.37) |
| SCS Isolation | 95 | 10.25 | (3.21) | 10.0 | 0.40 | 0.01 | (0.33) |
| SCS Mindfulness | 95 | 12.45 | (2.71) | 12.0 | -0.03 | -0.01 | (0.28) |
| SCS Overidentification | 95 | 10.05 | (3.15) | 10.0 | 0.12 | -0.50 | (0.32) |
| SCS Self-judgment | 95 | 12.74 | (3.99) | 13.0 | 0.25 | -0.46 | (0.41) |
| SCS Self-kindness | 95 | 14.12 | (3.41) | 15.0 | -0.06 | -0.54 | (0.35) |
| *Mid 7-day assessment for MEMI* | | | | | | | |
| DERS Awareness | 96 | 15.78 | (4.72) | 15.0 | 0.29 | -0.35 | (0.48) |
| DERS Clarity | 96 | 12.10 | (3.07) | 12.0 | -0.08 | -0.29 | (0.31) |
| DERS Goals | 96 | 15.57 | (4.32) | 15.0 | 0.01 | -0.36 | (0.44) |
| DERS Impulsivity | 96 | 14.29 | (4.88) | 14.0 | 0.62 | -0.08 | (0.50) |
| DERS Nonacceptance | 96 | 14.09 | (5.27) | 13.0 | 0.65 | -0.20 | (0.54) |
| DERS Strategy | 96 | 19.20 | (6.42) | 18.0 | 0.96 | 0.88 | (0.66) |
| SCS Common humanity | 96 | 12.22 | (3.70) | 12.0 | -0.04 | -0.22 | (0.38) |
| SCS Isolation | 96 | 11.51 | (3.81) | 12.0 | -0.08 | -0.57 | (0.39) |
| SCS Mindfulness | 96 | 12.30 | (3.09) | 12.0 | 0.15 | -0.25 | (0.32) |
| SCS Overidentification | 96 | 11.32 | (3.63) | 11.0 | 0.16 | -0.25 | (0.37) |
| SCS Self-judgment | 96 | 14.67 | (4.34) | 14.5 | 0.06 | -0.71 | (0.44) |
| SCS Self-kindness | 96 | 15.33 | (4.44) | 15.0 | 0.17 | -0.56 | (0.45) |
| *Mid 7-day assessment for SM* | | | | | | | |
| DERS Awareness | 95 | 15.78 | (4.13) | 16.0 | 0.22 | 0.01 | (0.42) |
| DERS Clarity | 95 | 11.69 | (3.42) | 11.0 | 0.69 | 0.72 | (0.35) |
| DERS Goals | 95 | 16.20 | (4.26) | 16.0 | 0.03 | -0.70 | (0.44) |
| DERS Impulsivity | 95 | 14.05 | (4.70) | 13.0 | 0.44 | -0.31 | (0.48) |
| DERS Nonacceptance | 95 | 14.74 | (5.07) | 14.0 | 0.35 | -0.58 | (0.52) |
| DERS Strategy | 95 | 20.26 | (6.30) | 19.0 | 0.62 | -0.16 | (0.65) |
| SCS Common humanity | 95 | 11.59 | (3.86) | 12.0 | 0.11 | -0.58 | (0.40) |
| SCS Isolation | 95 | 10.66 | (3.41) | 10.0 | 0.37 | -0.28 | (0.35) |
| SCS Mindfulness | 95 | 12.49 | (3.03) | 12.0 | -0.07 | 0.00 | (0.31) |
| SCS Overidentification | 95 | 11.07 | (2.97) | 11.0 | -0.20 | -0.19 | (0.30) |
| SCS Self-judgment | 95 | 14.03 | (4.10) | 14.0 | -0.19 | -0.61 | (0.42) |
| SCS Self-kindness | 95 | 14.36 | (3.81) | 14.0 | 0.47 | 0.17 | (0.39) |

DERS, difficulties in emotion regulation; SCS, self-compassion scale.

Table S3

*Parallel mediation analysis of difficulties in emotion regulation scale (DERS) and self-compassion scale (SCS) domains mediating the effect of group on various clinical outcomes from pre-post-intervention*

| *Difficulties in Emotion Regulation Scale (DERS) domains as the mediator of pre-post outcomes* | | | | |
| --- | --- | --- | --- | --- |
|  | β | *P* | *d* | [95% CI] |
| 1. *SAD symptoms (SPDQ score) as the outcome* | | | | |
| DERS Emotional awareness issues | -0.09 | .490 | -0.05 | [-0.19, 0.09] |
| DERS Lack of emotional clarity | 0.13 | .337 | 0.07 | [-0.08, 0.21] |
| DERS Goal-directed behavior difficulties | 0.03 | .789 | 0.02 | [-0.13, 0.16] |
| DERS Impulse control issues | -0.47 | .285 | -0.08 | [-0.22, 0.07] |
| DERS Non-acceptance of emotions | 0.00 | .901 | 0.01 | [-0.14, 0.15] |
| DERS Strategy usage problems | 0.00 | 1.000 | 0.00 | [-0.14, 0.14] |
| Total effect | -0.19 | .871 | -0.01 | [-0.16, 0.13] |
| 1. *SAD symptoms (SPIN score) as the outcome* | | | | |
| DERS Emotional awareness issues | -0.17 | .481 | -0.05 | [-0.20, 0.09] |
| DERS Lack of emotional clarity | 0.04 | .790 | 0.02 | [-0.13, 0.16] |
| DERS Goal-directed behavior difficulties | 0.06 | .788 | 0.02 | [-0.13, 0.16] |
| DERS Impulse control issues | -0.66 | .287 | -0.08 | [-0.22, 0.07] |
| DERS Non-acceptance of emotions | 0.03 | .893 | 0.01 | [-0.14, 0.15] |
| DERS Strategy usage problems | -0.20 | .420 | -0.06 | [-0.20, 0.09] |
| Total effect | 0.14 | .937 | 0.01 | [-0.14, 0.15] |
| 1. *Generalized anxiety symptoms (GADQ-IV score) as the outcome* | | | | |
| DERS Emotional awareness issues | 0.00 | .978 | 0.00 | [-0.14, 0.15] |
| DERS Lack of emotional clarity | -0.02 | .702 | -0.03 | [-0.17, 0.12] |
| DERS Goal-directed behavior difficulties | 0.01 | .788 | 0.02 | [-0.13, 0.16] |
| DERS Impulse control issues | -0.17 | .286 | -0.08 | [-0.22, 0.07] |
| DERS Non-acceptance of emotions | 0.00 | .897 | 0.01 | [-0.14, 0.15] |
| DERS Strategy usage problems | -0.06 | .412 | -0.06 | [-0.20, 0.09] |
| Total effect | 0.30 | .516 | 0.05 | [-0.10, 0.19] |
| 1. *Depression severity (BDI-II score) as the outcome* | | | | |
| DERS Emotional awareness issues | -0.27 | .461 | -0.05 | [-0.20, 0.09] |
| DERS Lack of emotional clarity | -0.06 | .646 | -0.03 | [-0.18, 0.11] |
| DERS Goal-directed behavior difficulties | -0.02 | .801 | -0.02 | [-0.16, 0.13] |
| DERS Impulse control issues | -0.57 | .287 | -0.08 | [-0.22, 0.07] |
| DERS Non-acceptance of emotions | 0.00 | .936 | 0.01 | [-0.14, 0.15] |
| DERS Strategy usage problems | -0.05 | .608 | -0.04 | [-0.18, 0.11] |
| Total effect | -1.72 | .259 | -0.08 | [-0.23, 0.06] |
| 1. *Trait repetitive negative thinking (PCQ score) as the outcome* | | | | |
| DERS Emotional awareness issues | -0.01 | .478 | -0.05 | [-0.20, 0.09] |
| DERS Lack of emotional clarity | -0.01 | .440 | -0.06 | [-0.20, 0.09] |
| DERS Goal-directed behavior difficulties | 0.00 | .792 | 0.02 | [-0.13, 0.16] |
| DERS Impulse control issues | -0.04 | .285 | -0.08 | [-0.22, 0.07] |
| DERS Non-acceptance of emotions | 0.00 | .896 | -0.01 | [-0.15, 0.14] |
| DERS Strategy usage problems | 0.00 | .955 | 0.00 | [-0.14, 0.15] |
| Total effect | -0.01 | .905 | -0.01 | [-0.15, 0.14] |
| 1. *Trait mindfulness (FFMQ total) as the outcome* | | | | |
| DERS Emotional awareness issues | 0.18 | .481 | 0.05 | [-0.09, 0.20] |
| DERS Lack of emotional clarity | 0.54 | .198 | 0.09 | [-0.05, 0.24] |
| DERS Goal-directed behavior difficulties | -0.03 | .792 | -0.02 | [-0.16, 0.13] |
| DERS Impulse control issues | 0.32 | .319 | 0.07 | [-0.07, 0.22] |
| DERS Non-acceptance of emotions | -0.07 | .893 | -0.01 | [-0.15, 0.13] |
| DERS Strategy usage problems | -0.04 | .697 | -0.03 | [-0.17, 0.12] |
| Total effect | 0.25 | .898 | 0.01 | [-0.14, 0.15] |
| *Self-Compassion Scale (SCS) domains as the mediator of pre-post outcomes* | | | | |
|  | β | *P* | *d* | [95% CI] |
| 1. *SAD symptoms (SPDQ score) as the outcome* | | | | |
| SCS Acknowledging shared human struggles | 0.04 | .719 | 0.03 | [-0.12, 0.17] |
| SCS Social connectedness | -0.13 | .404 | -0.06 | [-0.21, 0.08] |
| SCS Non-identification with emotions | -0.02 | .899 | -0.01 | [-0.15, 0.14] |
| SCS Mindfulness | -0.38 | .103 | -0.12 | [-0.26, 0.03] |
| SCS Non-judgment toward oneself | -0.18 | .346 | -0.07 | [-0.21, 0.08] |
| SCS Self-kindness | 0.02 | .871 | 0.01 | [-0.13, 0.16] |
| Total effect | -0.35 | .750 | -0.02 | [-0.17, 0.12] |
| 1. *SAD symptoms (SPIN score) as the outcome* | | | | |
| SCS Acknowledging shared human struggles | -0.23 | .285 | -0.08 | [-0.22, 0.07] |
| SCS Social connectedness | -0.05 | .629 | -0.03 | [-0.18, 0.11] |
| SCS Non-identification with emotions | 0.22 | .317 | 0.07 | [-0.07, 0.22] |
| SCS Mindfulness | -0.93 | .069 | -0.13 | [-0.28, 0.01] |
| SCS Non-judgment toward oneself | -0.35 | .333 | -0.07 | [-0.21, 0.07] |
| SCS Self-kindness | 0.03 | .871 | 0.01 | [-0.13, 0.16] |
| Total effect | -0.04 | .981 | 0.00 | [-0.15, 0.14] |
| 1. *Generalized anxiety symptoms (GADQ-IV score) as the outcome* | | | | |
| SCS Acknowledging shared human struggles | 0.06 | .300 | 0.08 | [-0.07, 0.22] |
| SCS Social connectedness | -0.06 | .393 | -0.06 | [-0.21, 0.08] |
| SCS Non-identification with emotions | -0.02 | .689 | -0.03 | [-0.17, 0.12] |
| SCS Mindfulness | -0.11 | .156 | -0.10 | [-0.25, 0.04] |
| SCS Non-judgment toward oneself | -0.06 | .364 | -0.07 | [-0.21, 0.08] |
| SCS Self-kindness | 0.01 | .871 | 0.01 | [-0.13, 0.16] |
| Total effect | 0.24 | .583 | 0.04 | [-0.10, 0.18] |
| 1. *Depression severity (BDI-II score) as the outcome* | | | | |
| SCS Acknowledging shared human struggles | 0.16 | .347 | 0.07 | [-0.08, 0.21] |
| SCS Social connectedness | -0.30 | .379 | -0.06 | [-0.21, 0.08] |
| SCS Non-identification with emotions | -0.16 | .394 | -0.06 | [-0.21, 0.08] |
| SCS Mindfulness | -0.21 | .321 | -0.07 | [-0.22, 0.07] |
| SCS Non-judgment toward oneself | -0.26 | .342 | -0.07 | [-0.21, 0.08] |
| SCS Self-kindness | 0.04 | .871 | 0.01 | [-0.13, 0.16] |
| Total effect | -1.93 | .199 | -0.09 | [-0.24, 0.05] |
| 1. *Trait repetitive negative thinking (PCQ score) as the outcome* | | | | |
| SCS Acknowledging shared human struggles | 0.00 | .623 | -0.04 | [-0.18, 0.11] |
| SCS Social connectedness | -0.01 | .391 | -0.06 | [-0.21, 0.08] |
| SCS Non-identification with emotions | 0.02 | .179 | 0.10 | [-0.05, 0.24] |
| SCS Mindfulness | -0.03 | .122 | -0.11 | [-0.26, 0.03] |
| SCS Non-judgment toward oneself | -0.03 | .320 | -0.07 | [-0.22, 0.07] |
| SCS Self-kindness | 0.00 | .871 | 0.01 | [-0.13, 0.16] |
| Total effect | -0.02 | .847 | -0.01 | [-0.16, 0.13] |
| 1. *Trait mindfulness (FFMQ total) as the outcome* | | | | |
| SCS Acknowledging shared human struggles | 0.59 | .180 | -0.05 | [-0.20, 0.09] |
| SCS Social connectedness | 0.06 | .642 | -0.06 | [-0.20, 0.09] |
| SCS Non-identification with emotions | -0.06 | .765 | 0.02 | [-0.13, 0.16] |
| SCS Mindfulness | 0.44 | .176 | -0.08 | [-0.22, 0.07] |
| SCS Non-judgment toward oneself | 0.47 | .328 | -0.01 | [-0.15, 0.14] |
| SCS Self-kindness | -0.03 | .872 | 0.00 | [-0.14, 0.15] |
| Total effect | 0.37 | .852 | -0.01 | [-0.15, 0.14] |

β, mediation regression estimate; *df*, degrees of freedom of the model; *p*, *p-*value associated with β; *d*, Cohen's *d* effect size; 95% CI, lower and upper bounds of the 95% confidence interval; SPDQ, social phobia diagnostic questionnaire; SPIN, social phobia inventory; GADQ-IV, generalized anxiety disorder questionnaire-fourth edition; BDI-II, Beck depression inventory-second edition; PCQ, perseverative cognitions questionnaire; FFMQ, five-facet mindfulness questionnaire.

Table S4

*Parallel mediation analysis of difficulties in emotion regulation scale (DERS) and self-compassion scale (SCS) domains mediating the effect of group on various clinical outcomes from pre-1-month follow-up (pre-1MFU)*

| *Difficulties in Emotion Regulation Scale (DERS) domains as the mediator of pre-1MFU outcomes* | | | | |
| --- | --- | --- | --- | --- |
|  | β | *P* | *d* | [95% CI] |
| 1. *SAD symptoms (SPDQ score) as the outcome* | | | | |
| DERS Emotional awareness issues | 0.00 | .976 | 0.00 | [-0.14, 0.15] |
| DERS Lack of emotional clarity | -0.01 | .939 | -0.01 | [-0.15, 0.14] |
| DERS Goal-directed behavior difficulties | 0.06 | .592 | 0.04 | [-0.11, 0.18] |
| DERS Impulse control issues | 0.01 | .805 | 0.02 | [-0.13, 0.16] |
| DERS Non-acceptance of emotions | -0.11 | .484 | -0.05 | [-0.2, 0.09] |
| DERS Strategy usage problems | -0.55 | .285 | -0.08 | [-0.22, 0.07] |
| Total effect | -0.35 | .779 | -0.02 | [-0.17, 0.12] |
| 1. *SAD symptoms (SPIN score) as the outcome* | | | | |
| DERS Emotional awareness issues | 0.01 | .897 | 0.01 | [-0.14, 0.15] |
| DERS Lack of emotional clarity | -0.09 | .528 | -0.05 | [-0.19, 0.10] |
| DERS Goal-directed behavior difficulties | 0.29 | .274 | 0.08 | [-0.07, 0.22] |
| DERS Impulse control issues | 0.07 | .787 | 0.02 | [-0.13, 0.16] |
| DERS Non-acceptance of emotions | -0.11 | .527 | -0.05 | [-0.19, 0.10] |
| DERS Strategy usage problems | -0.89 | .284 | -0.08 | [-0.22, 0.07] |
| Total effect | 1.60 | .422 | 0.06 | [-0.09, 0.20] |
| 1. *Generalized anxiety symptoms (GADQ-IV score) as the outcome* | | | | |
| DERS Emotional awareness issues | 0.00 | .906 | 0.01 | [-0.14, 0.15] |
| DERS Lack of emotional clarity | -0.04 | .449 | -0.05 | [-0.2, 0.09] |
| DERS Goal-directed behavior difficulties | -0.03 | .479 | -0.05 | [-0.2, 0.09] |
| DERS Impulse control issues | 0.00 | .917 | 0.01 | [-0.14, 0.15] |
| DERS Non-acceptance of emotions | -0.01 | .674 | -0.03 | [-0.18, 0.11] |
| DERS Strategy usage problems | -0.19 | .286 | -0.08 | [-0.22, 0.07] |
| Total effect | 0.38 | .418 | 0.06 | [-0.09, 0.20] |
| 1. *Depression severity (BDI-II score) as the outcome* | | | | |
| DERS Emotional awareness issues | -0.01 | .895 | -0.01 | [-0.15, 0.14] |
| DERS Lack of emotional clarity | -0.03 | .734 | -0.02 | [-0.17, 0.12] |
| DERS Goal-directed behavior difficulties | 0.08 | .564 | 0.04 | [-0.1, 0.19] |
| DERS Impulse control issues | -0.02 | .799 | -0.02 | [-0.16, 0.13] |
| DERS Non-acceptance of emotions | -0.29 | .460 | -0.05 | [-0.20, 0.09] |
| DERS Strategy usage problems | -0.60 | .287 | -0.08 | [-0.22, 0.07] |
| Total effect | -1.26 | .429 | -0.06 | [-0.20, 0.09] |
| 1. *Trait repetitive negative thinking (PCQ score) as the outcome* | | | | |
| DERS Emotional awareness issues | 0.00 | .896 | 0.01 | [-0.14, 0.15] |
| DERS Lack of emotional clarity | -0.01 | .441 | -0.06 | [-0.20, 0.09] |
| DERS Goal-directed behavior difficulties | -0.01 | .572 | -0.04 | [-0.19, 0.1] |
| DERS Impulse control issues | 0.00 | .827 | -0.02 | [-0.16, 0.13] |
| DERS Non-acceptance of emotions | -0.01 | .470 | -0.05 | [-0.2, 0.09] |
| DERS Strategy usage problems | -0.06 | .283 | -0.08 | [-0.22, 0.07] |
| Total effect | 0.05 | .671 | 0.03 | [-0.11, 0.18] |
| 1. *Trait mindfulness (FFMQ total) as the outcome* | | | | |
| DERS Emotional awareness issues | -0.07 | .893 | -0.01 | [-0.15, 0.13] |
| DERS Lack of emotional clarity | -0.19 | .437 | -0.06 | [-0.20, 0.09] |
| DERS Goal-directed behavior difficulties | 0.31 | .276 | 0.08 | [-0.07, 0.22] |
| DERS Impulse control issues | 0.01 | .846 | 0.01 | [-0.13, 0.16] |
| DERS Non-acceptance of emotions | 0.13 | .515 | 0.05 | [-0.1, 0.19] |
| DERS Strategy usage problems | 0.50 | .299 | 0.08 | [-0.07, 0.22] |
| Total effect | 0.85 | .685 | 0.03 | [-0.12, 0.17] |
| *Self-Compassion Scale (SCS) domains as the mediator of pre-1MFU outcomes* | | | | |
|  | β | *P* | *d* | [95% CI] |
| 1. *SAD symptoms (SPDQ score) as the outcome* | | | | |
| SCS Acknowledging shared human struggles | -0.07 | .598 | -0.04 | [-0.18, 0.11] |
| SCS Social connectedness | -0.32 | .136 | -0.11 | [-0.25, 0.04] |
| SCS Non-identification with emotions | -0.31 | .325 | -0.07 | [-0.22, 0.07] |
| SCS Mindfulness | 0.03 | .871 | 0.01 | [-0.13, 0.16] |
| SCS Non-judgment toward oneself | -0.16 | .392 | -0.06 | [-0.21, 0.08] |
| SCS Self-kindness | 0.09 | .428 | 0.06 | [-0.09, 0.20] |
| Total effect | -0.57 | .629 | -0.03 | [-0.18, 0.11] |
| 1. *SAD symptoms (SPIN score) as the outcome* | | | | |
| SCS Acknowledging shared human struggles | -0.14 | .522 | -0.05 | [-0.19, 0.10] |
| SCS Social connectedness | -0.62 | .110 | -0.12 | [-0.26, 0.03] |
| SCS Non-identification with emotions | -0.34 | .341 | -0.07 | [-0.21, 0.08] |
| SCS Mindfulness | 0.05 | .871 | 0.01 | [-0.13, 0.16] |
| SCS Non-judgment toward oneself | -0.26 | .395 | -0.06 | [-0.21, 0.08] |
| SCS Self-kindness | 0.28 | .266 | 0.08 | [-0.06, 0.23] |
| Total effect | 1.23 | .517 | 0.05 | [-0.10, 0.19] |
| 1. *Generalized anxiety symptoms (GADQ-IV score) as the outcome* | | | | |
| SCS Acknowledging shared human struggles | -0.05 | .396 | 0.01 | [-0.14, 0.15] |
| SCS Social connectedness | -0.14 | .120 | -0.08 | [-0.22, 0.07] |
| SCS Non-identification with emotions | -0.07 | .356 | 0.01 | [-0.14, 0.15] |
| SCS Mindfulness | 0.01 | .871 | -0.05 | [-0.2, 0.09] |
| SCS Non-judgment toward oneself | -0.06 | .397 | -0.05 | [-0.2, 0.09] |
| SCS Self-kindness | 0.06 | .279 | -0.03 | [-0.18, 0.11] |
| Total effect | 0.34 | .458 | 0.06 | [-0.09, 0.20] |
| 1. *Depression severity (BDI-II score) as the outcome* | | | | |
| SCS Acknowledging shared human struggles | -0.12 | .496 | -0.05 | [-0.19, 0.1] |
| SCS Social connectedness | -0.38 | .157 | -0.10 | [-0.25, 0.04] |
| SCS Non-identification with emotions | -0.02 | .876 | -0.01 | [-0.16, 0.13] |
| SCS Mindfulness | 0.05 | .871 | 0.01 | [-0.13, 0.16] |
| SCS Non-judgment toward oneself | -0.41 | .373 | -0.06 | [-0.21, 0.08] |
| SCS Self-kindness | 0.32 | .216 | 0.09 | [-0.06, 0.23] |
| Total effect | -1.45 | .365 | -0.07 | [-0.21, 0.08] |
| 1. *Trait repetitive negative thinking (PCQ score) as the outcome* | | | | |
| SCS Acknowledging shared human struggles | -0.02 | .191 | -0.09 | [-0.18, 0.11] |
| SCS Social connectedness | -0.01 | .367 | -0.07 | [-0.2, 0.09] |
| SCS Non-identification with emotions | -0.02 | .336 | -0.07 | [-0.14, 0.15] |
| SCS Mindfulness | 0.00 | .871 | 0.01 | [-0.22, 0.07] |
| SCS Non-judgment toward oneself | -0.03 | .373 | -0.06 | [-0.14, 0.15] |
| SCS Self-kindness | 0.02 | .252 | 0.08 | [-0.2, 0.09] |
| Total effect | 0.03 | .784 | 0.02 | [-0.09, 0.20] |
| 1. *Trait mindfulness (FFMQ total) as the outcome* | | | | |
| SCS Acknowledging shared human struggles | 0.30 | .279 | 0.08 | [-0.07, 0.22] |
| SCS Social connectedness | 0.53 | .148 | 0.10 | [-0.04, 0.25] |
| SCS Non-identification with emotions | 0.33 | .349 | 0.07 | [-0.08, 0.21] |
| SCS Mindfulness | -0.01 | .876 | -0.01 | [-0.16, 0.13] |
| SCS Non-judgment toward oneself | 0.00 | .990 | 0.00 | [-0.15, 0.14] |
| SCS Self-kindness | 0.68 | .175 | 0.10 | [-0.05, 0.24] |
| Total effect | 1.02 | .621 | 0.04 | [-0.11, 0.18] |

β, mediation regression estimate; *df*, degrees of freedom of the model; *p*, *p-*value associated with β; *d*, Cohen's *d* effect size; 95% CI, lower and upper bounds of the 95% confidence interval; SPDQ, social phobia diagnostic questionnaire; SPIN, social phobia inventory; GADQ-IV, generalized anxiety disorder questionnaire-fourth edition; BDI-II, Beck depression inventory-second edition; PCQ, perseverative cognitions questionnaire; FFMQ, five-facet mindfulness questionnaire.

**App intervention arms**

**Mindfulness ecological momentary intervention (MEMI).** Multimedia Appendix 1 displays screenshots of MEMI prompts. During each app prompt, MEMI participants were initially asked to engage in *slowed, steady, rhythmic breathing*, "Pay attention to your breathing. Breathe in a slow, steady, and rhythmic manner. Stay focused on the sensations of the air coming into your lungs and then letting it out. Click 'Continue'". Then, they were asked to exercise *open monitoring* and *acceptance*, "As you're breathing, observe your experience as it is. Let go of judgments that do not serve you. Focus your attention in the here-and-now. Click 'Continue.'" Finally, they were asked to *attend to small moments*: "Attend to the small moments right now (e.g., reading a chapter, having a cool glass of water) as that is where enjoyment, peace, and serenity in life happen. Click 'Okay' to continue."

**Self-monitoring app (SM).** Multimedia Appendix 1 shows screenshots of the app prompts for the SN condition. During each app prompt, SM participants were asked to observe their thoughts, "Notice your thoughts and how distressing they may be. Click 'Okay' to continue."

## Screenshots for mindfulness ecological momentary intervention (MEMI) arm


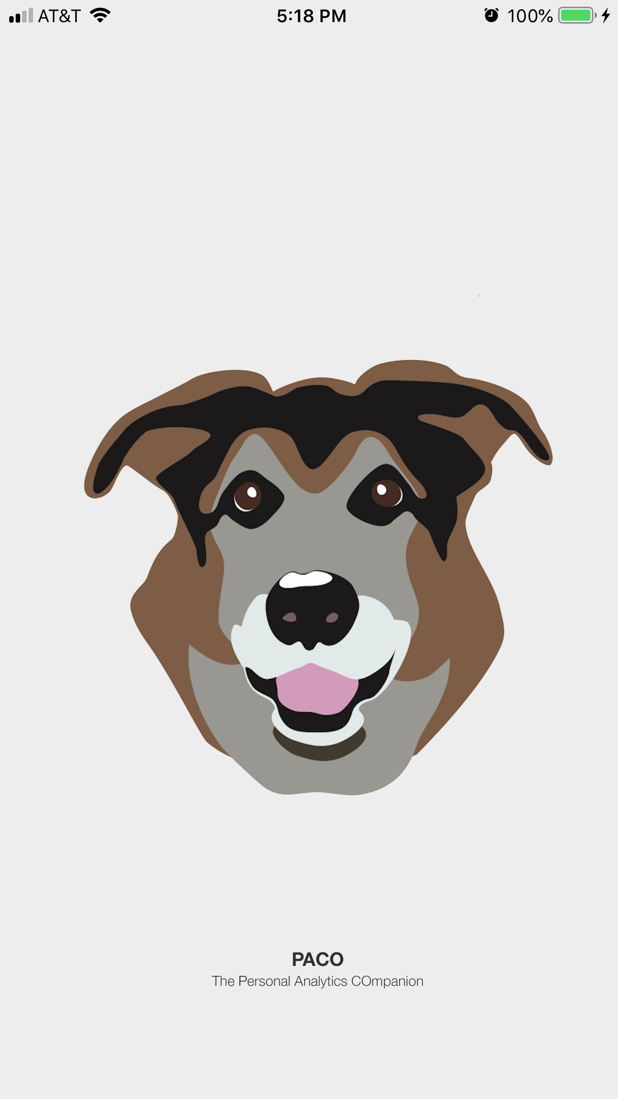

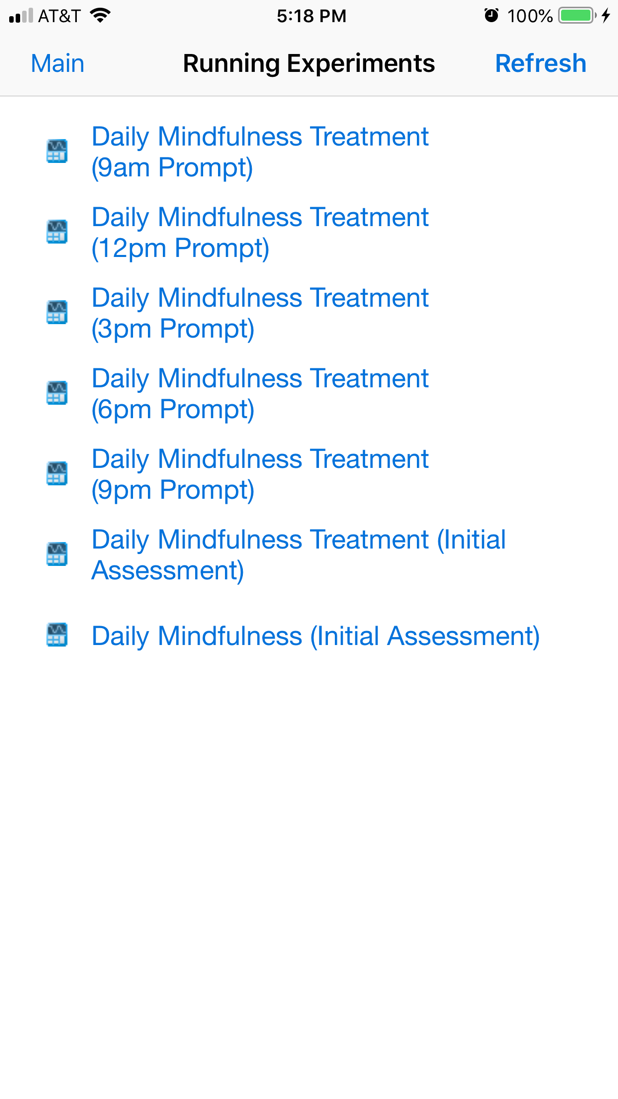


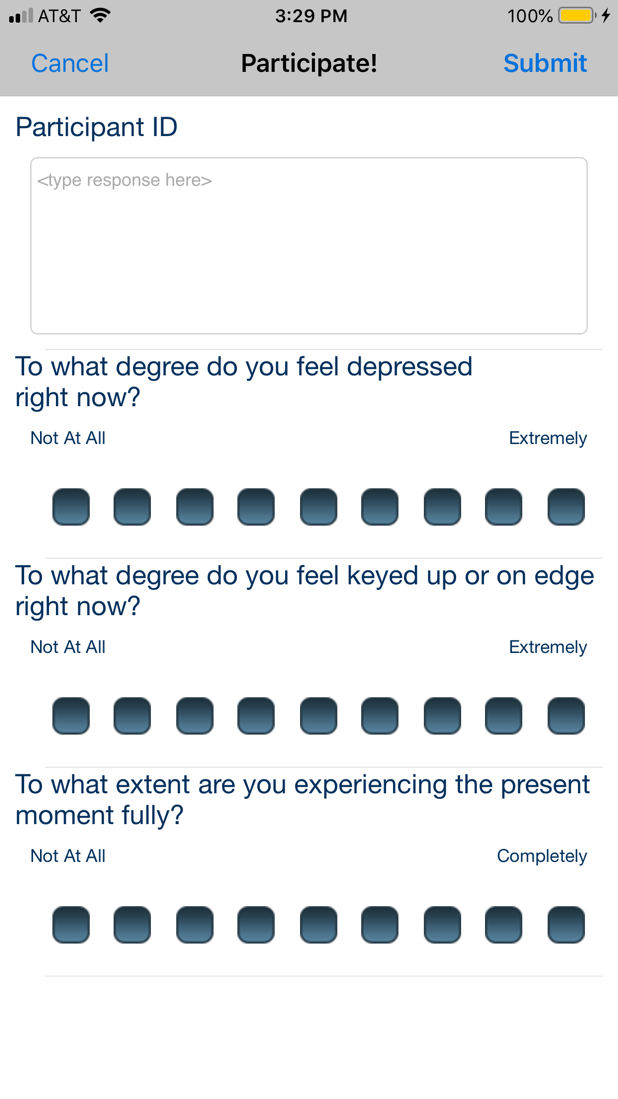

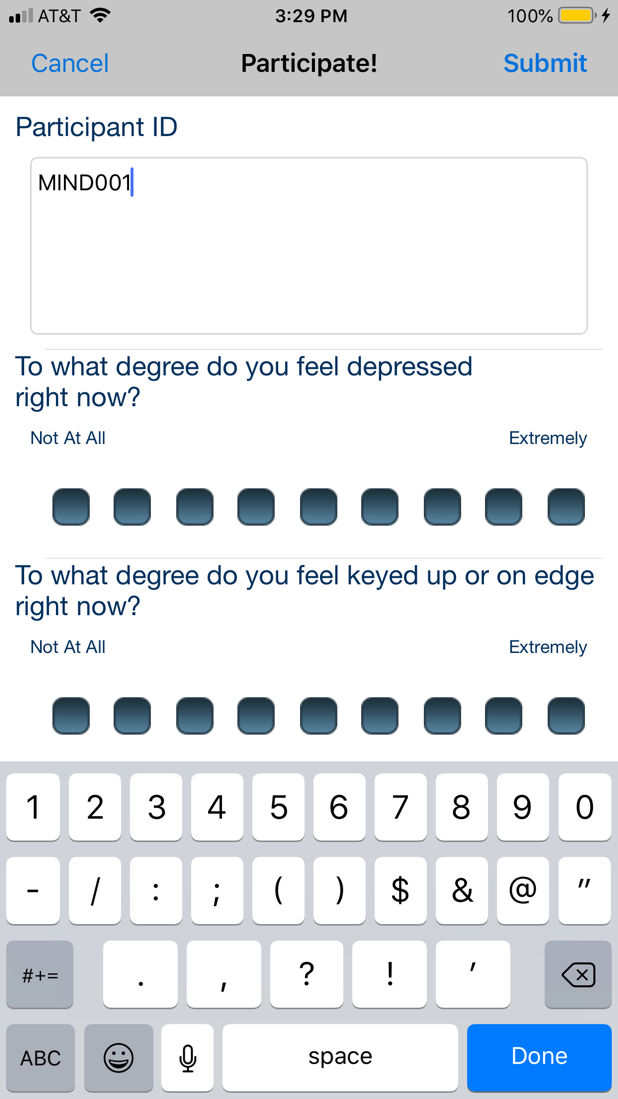


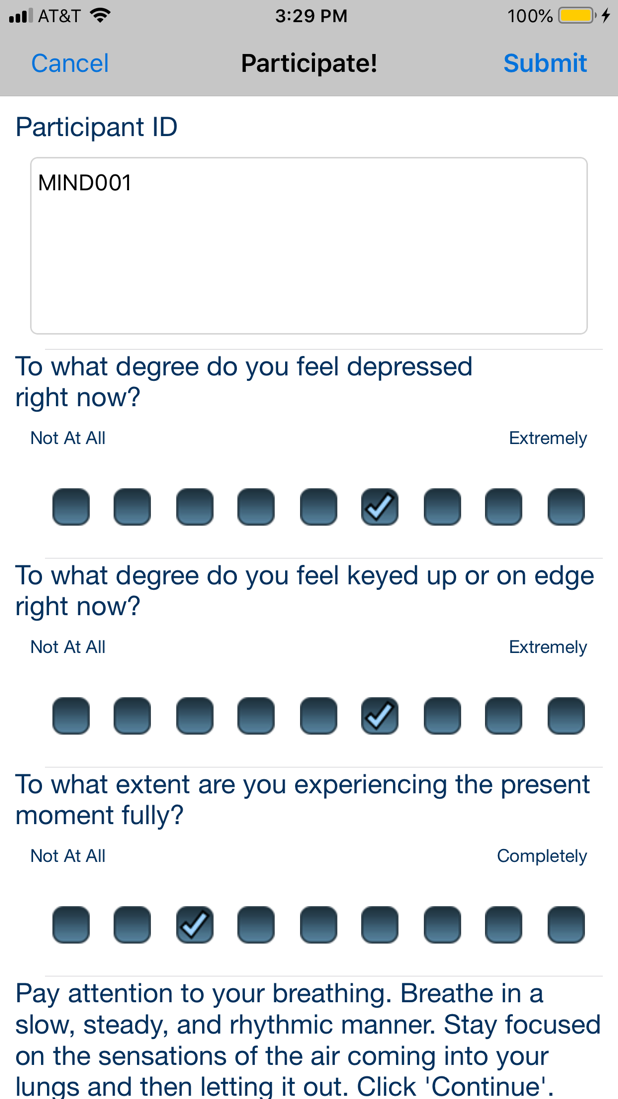

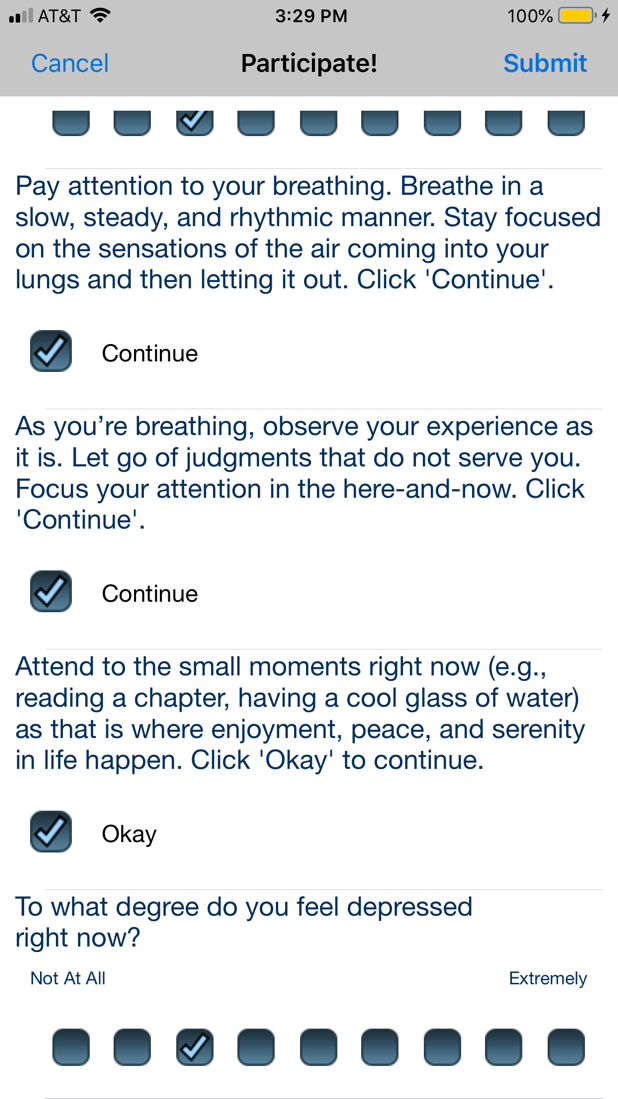


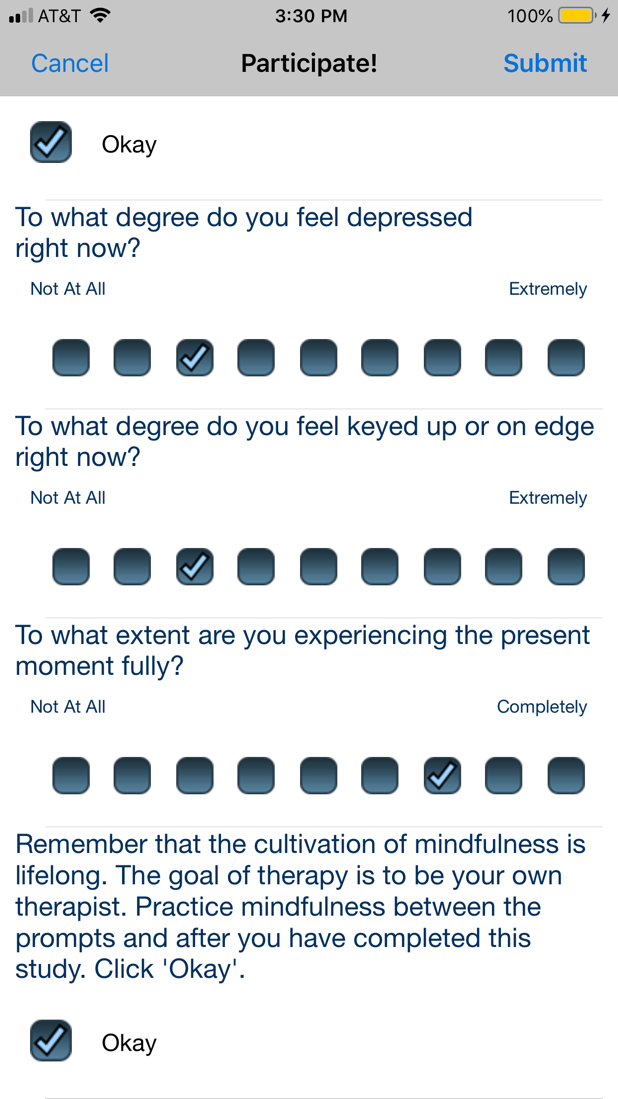

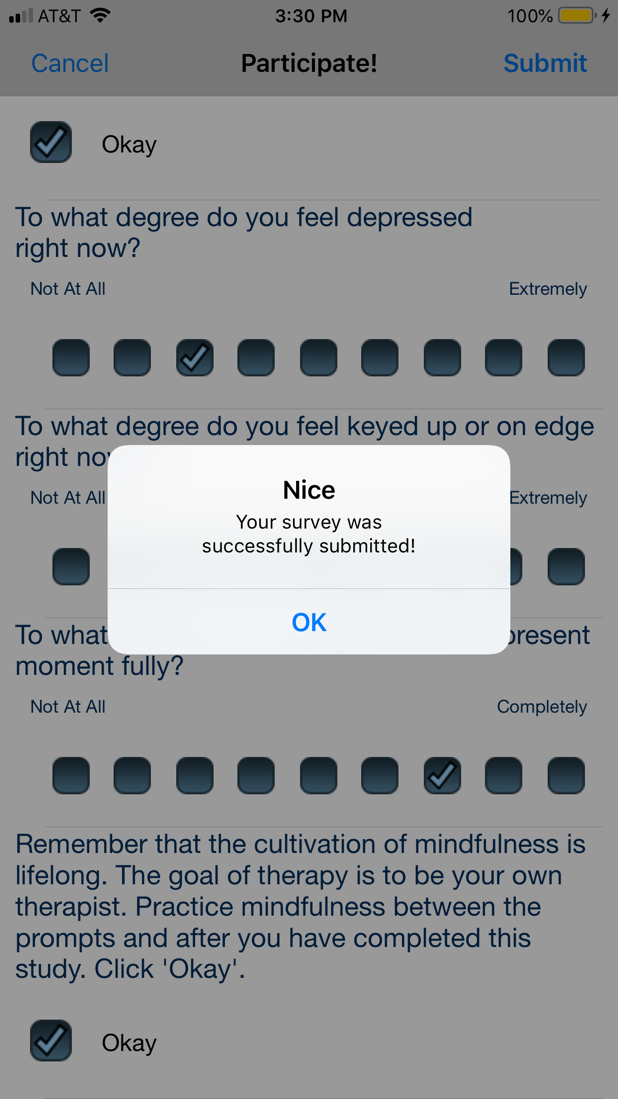


## Screenshots for self-monitoring app (SM) arm


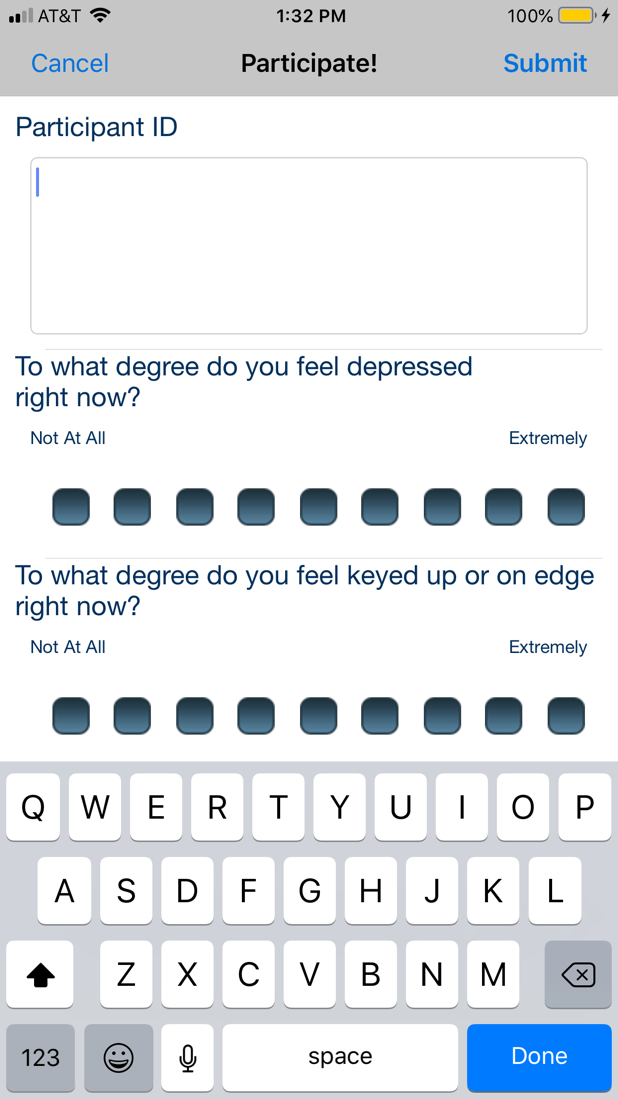

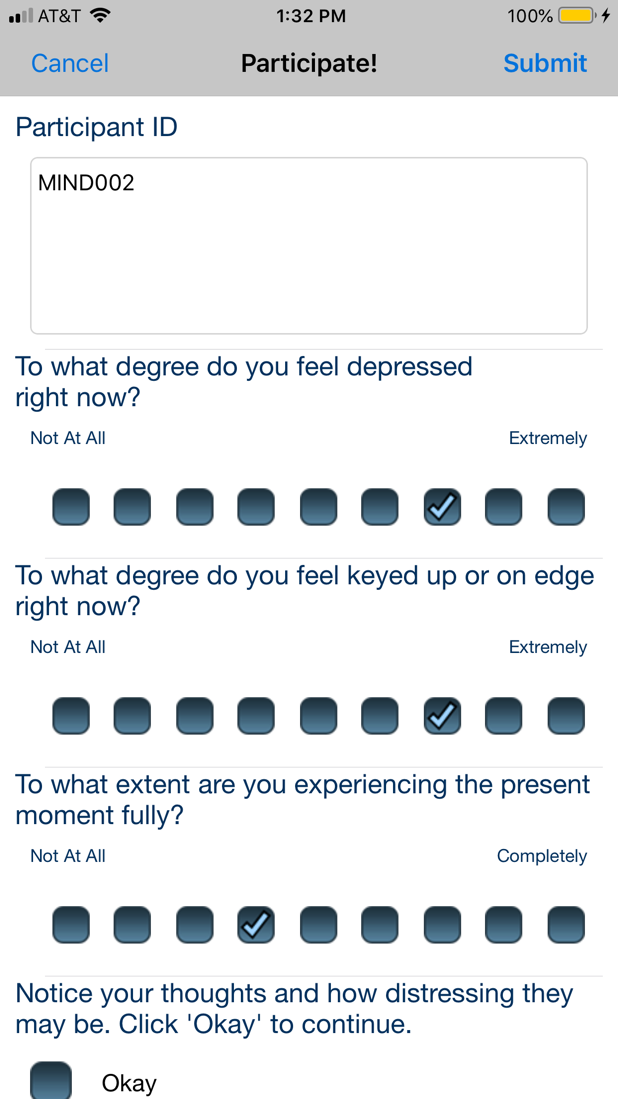


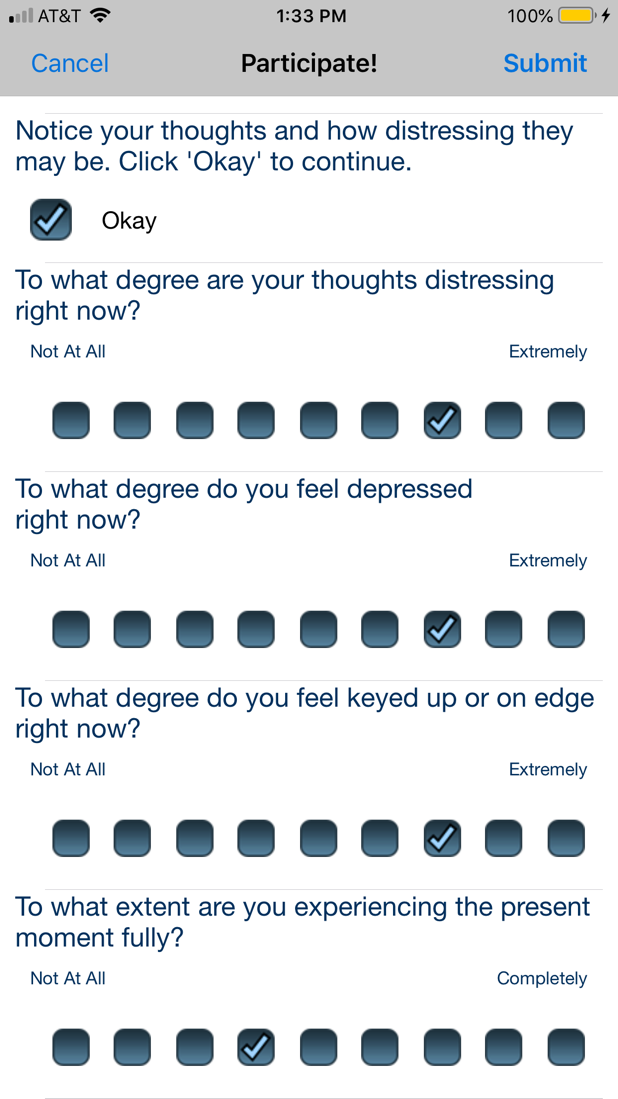

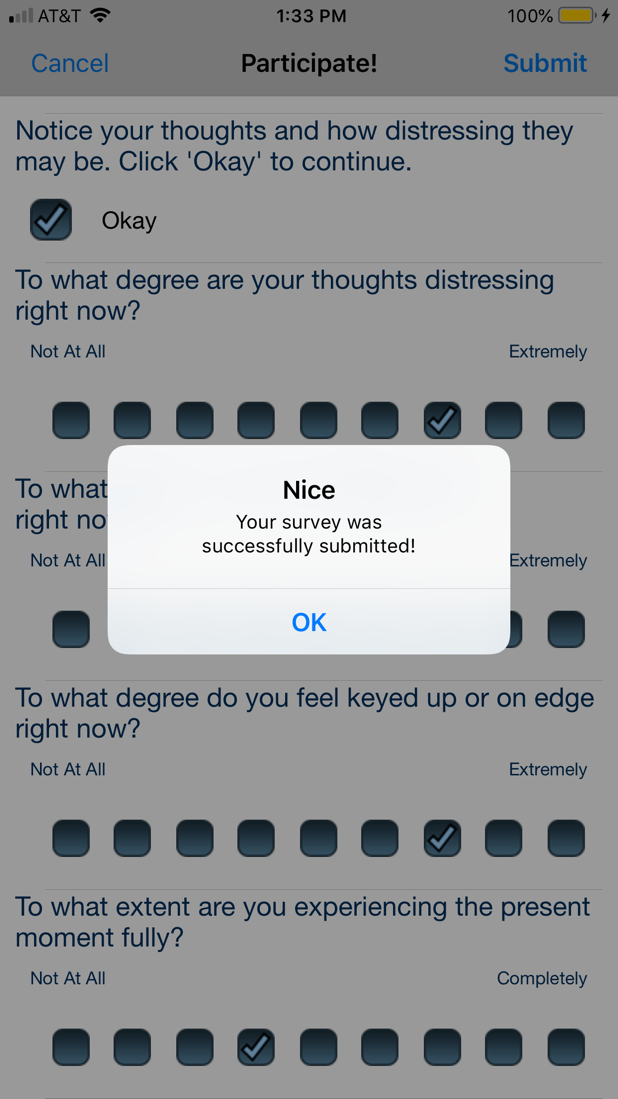

Supplement: Multimedia Appendix 1 [file mental_v11i1e53712_app1.docx]
